# Amidoxime‐Functionalized *sp* ^2^‐Carbon‐Conjugated Covalent Organic Frameworks for Overall Photocatalytic Hydrogen Peroxide Production

**DOI:** 10.1002/advs.202415194

**Published:** 2025-02-18

**Authors:** Zhiwu Yu, Fengtao Yu, Mei Xu, Shufan Feng, Jianding Qiu, Jianli Hua

**Affiliations:** ^1^ Key Laboratory for Advanced Materials and Joint International Research Laboratory for Precision Chemistry and Molecular Engineering Feringa Nobel Prize Scientist Joint Research Center Frontiers Science Center for Materiobiology and Dynamic Chemistry School of Chemistry and Molecular Engineering East China University of Science and Technology Meilong Road 130 Shanghai 200237 China; ^2^ State Key Laboratory of Nuclear Resources and Environment East China University of Technology GuangLan Avenue 418 Nanchang 330013 China

**Keywords:** amidoxime‐functionalized, artificial photosynthesis, charge separation, covalent organic frameworks, hydrogen peroxide

## Abstract

Cyano‐functionalized *sp* ^2^‐carbon‐conjugated covalent organic frameworks (CN‐COFs) have been considered as promising candidates for artificial photosynthesis of hydrogen peroxide (H_2_O_2_). Nevertheless, the performance of CN‐COFs is inherently limited by constrained oxygen capture capacity, insufficient charge separation, and rapid carrier recombination. Herein, the study rationally reports a strategy for integrating amidoxime groups (AO) into a COF through one‐step cyano hydrolysis process to increase photocatalytic H_2_O_2_ production. Combined simulations and characterizations reveal that introducing AO groups enhances hydrophilicity, stabilizes adsorbed Oxygen (O_2_) via hydrogen bonding, accelerates the charge separation and transfer, as well as lowers the energy barrier for oxygen reduction reaction pathway, thus achieving an unmatched H_2_O_2_ production rate of 6024 µmol h^−1^ g^−1^. Importantly, the solar‐to‐chemical conversion (SCC) efficiency of PTTN‐AO reaches 0.61%, significantly surpassing that of natural plants (≈0.1%) and most COF‐based photocatalysts. The current findings are encouraging for the molecular design of polymers for green and efficient H_2_O_2_ production.

## Graphical abstract

Amidoxime‐functionalization *sp* ^2^‐carbon‐conjugated organic framework (PTTN‐AO) is carefully designed and synthesized to increase hydrophilicity, stabilize adsorbed O_2_ via hydrogen bonding, and accelerate the charge separation and transfer. This strategy promotes the photocatalytic 2e^−^ oxygen reduction reaction to produce hydrogen peroxide.

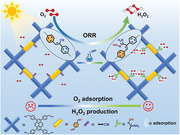

## 1 Introduction

Hydrogen peroxide, as an indispensable inorganic chemical, has been widely used in industrial manufacturing, sterilization, disinfection, and chemical synthesis due to its strong oxidative properties.^[^ ^1^ ^]^ Mature methods of production H₂O₂ are mainly focused on the environmentally unfriendly anthraquinone process, which require high‐pressure hydrogen and precious metal catalysts, as well as produce toxic byproducts and consuming substantial energy.^[^ ^2^ ^]^ An alternative promising strategy for green and efficient synthesizing H_2_O_2_ is sunlight‐driven photocatalytic two‐electron oxygen reduction reaction (2e^−^ ORR) via the naturally abundant water and air.^[^ ^3^ ^]^ Traditional metal‐based inorganic semiconductor photocatalysts have witnessed the vigorous development of artificial photosynthesis in recent years, while metal elements release, low selectivity, and decomposition of H_2_O_2_ during the photocatalytic process severely limit the practical application of such materials.^[^ ^4^ ^]^

Interestingly, conjugated organic polymer semiconductors have recently been recognized as green, safe, and low‐cost photocatalyst with wide‐ranging applications.^[^ ^5^ ^]^ Particularly, COFs, a novel subset of metal‐free *π*‐conjugated organic polymer photocatalysts, have garnered important attention for photocatalytic H₂O₂ synthesis, driven by their unique advantages, such as tunable building blocks, ordered structures, chemical stability, and abundant porosity.^[^ ^6^ ^]^ In parallel, there is growing interest in application of post‐synthetic modification (PSM) COFs for photocatalysis, as it provides the flexibility to introduce various functional groups without compromising the regularity of their parent counterparts, which makes this approach more suitable for expanding and enhancing COFs functionality.^[^ ^7^ ^]^ Given its promotion of charge carrier separation, reduced recombination, lowered energy barrier, and stable O₂ adsorption, PSM‐COFs have essential advantages in enhancing the photocatalytic H_2_O_2_ production. Particularly, the cyano‐functionalized COFs play a considerable role in effectively enhancing light absorption and photogenerated electron transport.^[^ ^8^ ^]^ Therefore, many studies have introduced CN groups into COFs to elevate their activity for photocatalytic H₂O₂ production. For instance, Han et al.^[^ ^9^ ^]^ incorporated CN groups into imine‐linked COFs, promoting the conversion of O₂ to ^1^O₂, thereby accelerating the 2e^−^ ORR pathway for H₂O₂ production. Similarly, Tong et al.^[^ ^10^ ^]^ adjusted the number of CN groups in COF side chains to control the number of photogenerated electron transport channels, finding that dicyano‐functionalized COFs exhibited the highest efficiency in charge separation and transport. However, despite the role of CN groups in facilitating O_2_ reduction to H₂O₂ in these COFs, its poor hydrophilicity and weak oxygen capture ability limit its overall effectiveness for photocatalytic H₂O₂ production, which hampering the practical application. Even worse, toxic CN units are harmful to the environment and humans and do not comply with the principles of green chemistry. Accordingly, there is an urgent need to explore new COFs modified with more active and non‐toxic organic groups to achieve green and efficient H_2_O_2_ production, but this is full of challenges.

Amidoxime‐functionalization COFs (AO‐COFs) which obtained through one‐step hydrolysis of CN groups have been valid used for uranium extraction from seawater.^[^ ^11^ ^]^ In addition to its unique uranium affinity, there is evidence to suggest that the introduction of AO groups considerably enhances the hydrophilicity of COFs, improves the adsorption efficiency of water and oxygen, as well as accelerate charge carrier separation, which will be very beneficial for photocatalytic reactions involving oxygen reduction and water oxidation.^[^ ^12^ ^]^ Regrettably, the application of AO‐COF in photocatalytic H_2_O_2_ production is still blank so far. Therefore, we reasonably speculate that AO‐COFs will have the potential to replace CN‐COF and become a star candidate in the field of photocatalytic H_2_O_2_ production.

As a proof of concept, herein, a novel *sp* ^2^‐carbon‐conjugated COF named PTTN‐CN with donor‐acceptor skeleton containing CN groups has been synthesized via the Knoevenagel condensation reaction; then AO groups were introduced via a post‐functionalized strategy to obtain another COF named PTTN‐AO. Using water and O_2_ as reactants, we evaluated the photocatalytic performance of both COFs for H₂O₂ production (Figure S1, Supporting Information). The incorporation of AO groups not only optimized charge distribution, but facilitated proton transfer and stabilized O₂ adsorption via hydrogen bonding, thus leading to a markedly higher H₂O₂ production rate of 6024 µmol g⁻¹ h⁻¹ under visible light irradiation. Excitingly, a recorded SCC efficiency of 0.61% has been achieved. Moreover, a comprehensive investigation into the mechanisms and reaction pathways of the two COFs was conducted through a combination of various experiments and theoretical calculations.

## 2 Results and Discussion

**Figure 1:**
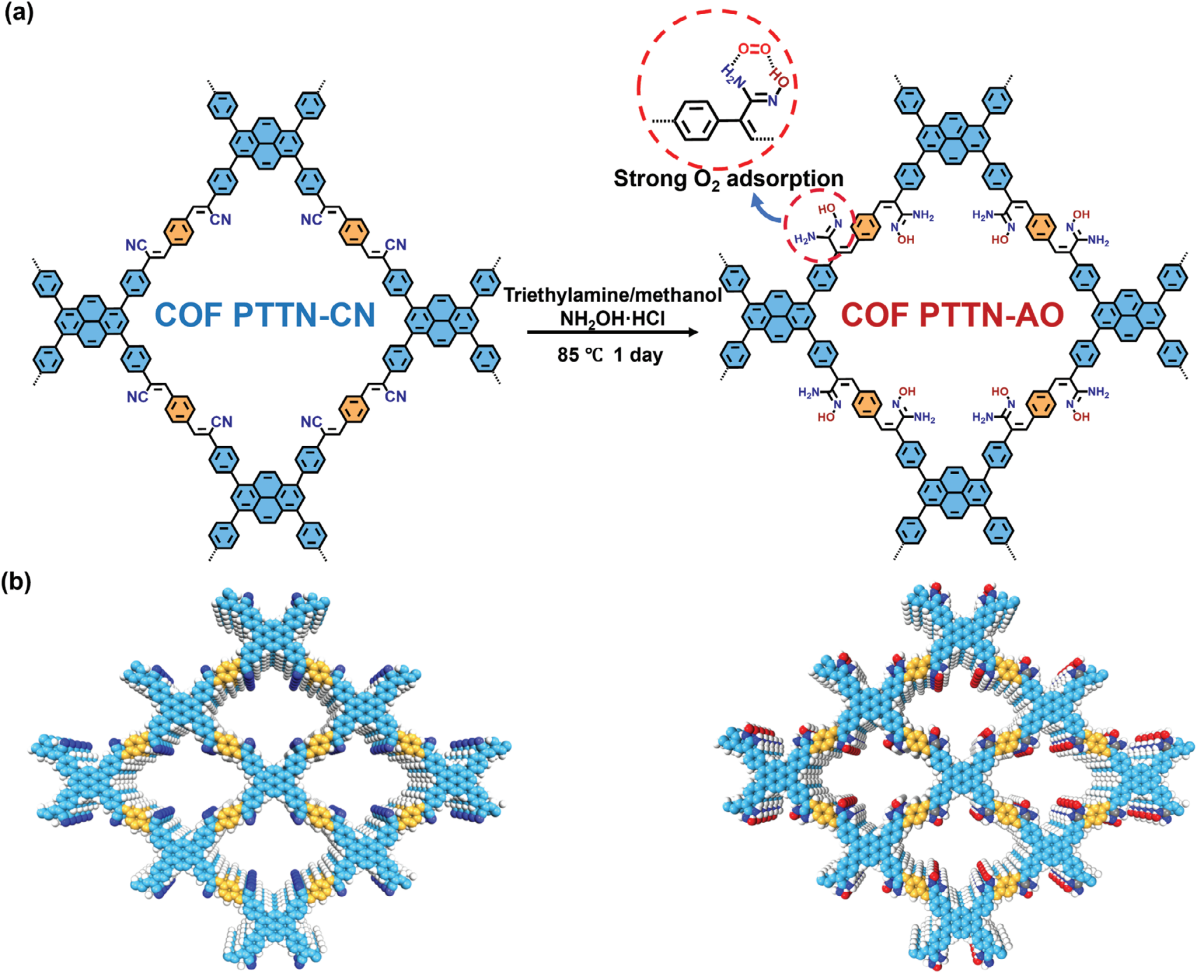
a) Synthesis of PTTN‐AO; b) AA stacking mode of PTTN‐CN and PTTN‐AO.

**Figure 2:**
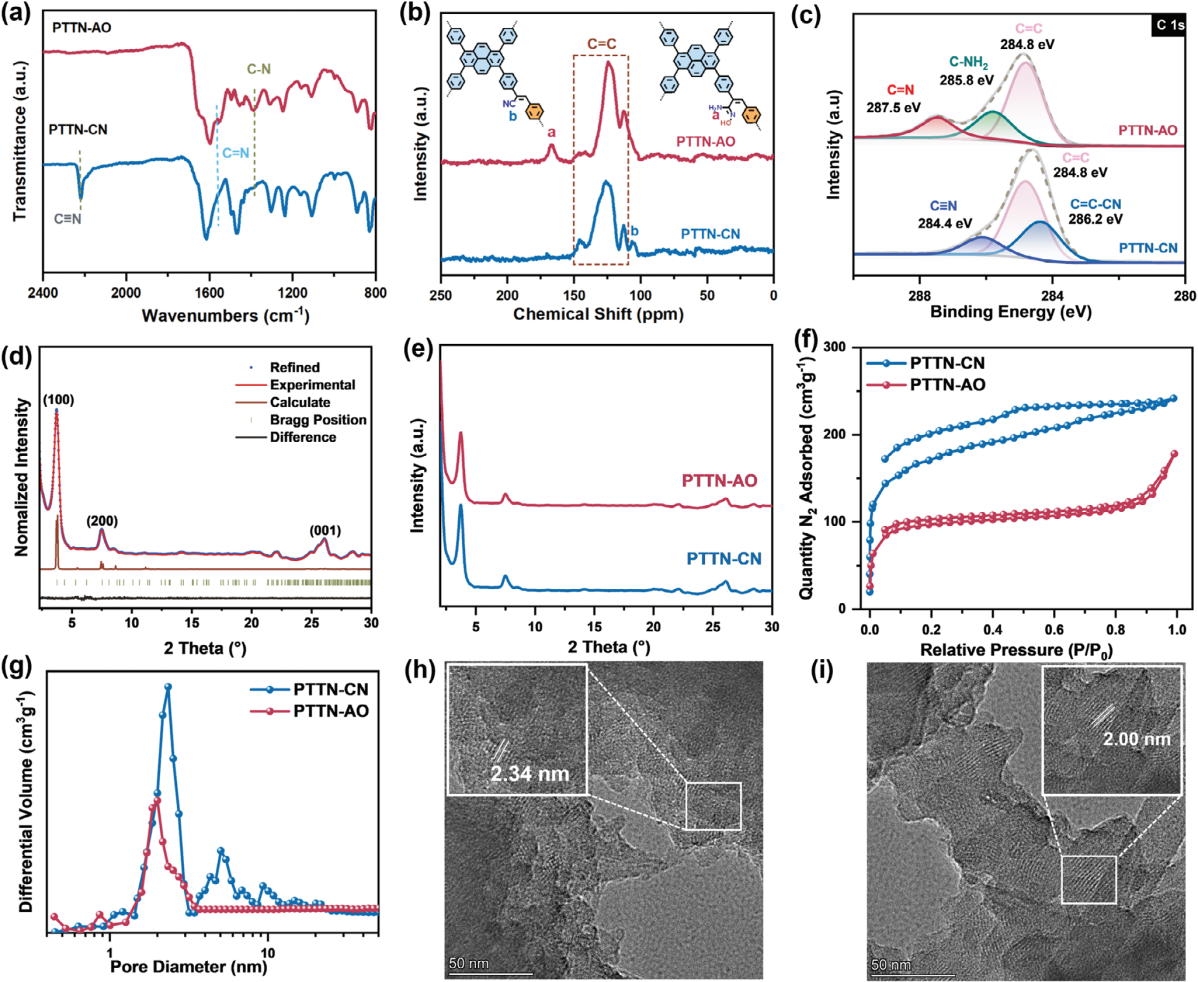
a) FT‐IR spectrum of PTTN‐CN and PTTN‐AO. b) Solid‐state ^13^C CP/MAS‐NMR spectra of PTTN‐CN and PTTN‐AO. c) The XPS spectra of C 1s for PTTN‐CN and PTTN‐AO. d) PXRD patterns of PTTN‐CN. e) PXRD patterns of PTTN‐CN and PTTN‐AO. f) N_2_ adsorption‐desorption isotherms at 77.3 K for PTTN‐CN and PTTN‐AO. g) Pore size distribution for PTTN‐CN and PTTN‐AO. e,f) HR‐TEM image of PTTN‐CN and PTTN‐AO.

As described in Supporting Information S1.13, PTTN‐CN was innovatively synthesized via a Knoevenagel polycondensation reaction between 4,4′,4″,4′″‐(1,3,6,8‐pyrenetetrayl)‐tetrabenzonitrile (PTTN) and 1,4‐phthalaldehyde. Then, PTTN‐CN was reacted with triethylamine and hydroxylamine hydrochloride (NH_2_OH·HCl) at 85 °C for 1 day to obtain the AO‐modified COF named PTTN‐AO (**Figure** **1**a,b). The chemical structure of the as‐synthesized COFs was thoroughly characterized by Fourier‐transform infrared (FT‐IR) spectroscopy, solid‐state ¹^3^C nuclear magnetic resonance (¹^3^C NMR) spectroscopy, and X‐ray photoelectron spectroscopy (XPS). According to FT‐IR spectrum of PTTN‐CN, the disappearance of the ‐CHO stretching vibration peak (1696 cm⁻¹) and the shift of the preserved C≡N vibration peak from 2250 cm⁻¹ in Py‐CN to 2217 cm⁻¹ in PTTN‐CN (**Figure** **2**a; Figure S2, Supporting Information), confirming that C═C was formed through Knoevenagel condensation. For PTTN‐AO, the characteristic C≡N signal disappear entirely, and new stretching vibration peaks appear at 1562 and 1385 cm⁻¹, corresponding to C═N and C–N in the AO group, suggesting that success of transformation from PTTN‐CN to PTTN‐AO (Figure 2a). The solid‐state ^13^C NMR, depicted in Figure 2b, revealed the disappearance of signal at 106 ppm for the C≡N and the appearance of a new peak at 166 ppm for C = N, validating the complete conversion of CN to AO groups, which was consistent with the FT‐IR results. High‐resolution XPS measurements demonstrated that the C 1s peak of PTTN‐CN could be deconvoluted into sp‐hybridized carbon (C≡N) and sp^2^‐hybridized carbon (C ═ C–CN) at 286.2 and 284.4 eV (Figure 2c), indicating the successful introduction of CN into PTTN‐CN. For C 1s peak of PTTN‐AO, new signals at 285.8 and 287.5 eV were clearly observed, corresponding to amino‐linked carbon (C = NH_2_) and imine bonds (C ═ N), respectively.

The crystallinity of PTTN‐CN was determined by experimental powder X‐ray diffraction (PXRD) combined with theoretical simulations. As shown in Figure 2d, the diffraction peaks of PTTN‐CN at 2*θ* ≈3.72°, 7.50°, and 26.08° correspond to the (100), (200), and (001) planes, respectively. The optimized structures of PTTN‐CN belonged to the *P1* space group with refined cell parameters of a = 3.66 Å, b = 23.37 Å, c = 23.83 Å, 𝛼 = 86.47°, 𝛽 = 89.00°, and 𝛾 = 91.91°, and the negligible *R_wp_* (4.38%) and *R_p_* (3.88%) values prove a high degree of agreement between the theoretical model (eclipsed AA stacking mode) and the experimental structure (Figure S4 and Table S2, Supporting Information). Notably, despite the grafting of AO disrupting the microenvironment, PTTN‐AO exhibited the similar PXRD pattern as PTTN‐CN (Figure 2e). This result indicates that the crystallinity of PTTN‐AO was well‐preserved after the post‐modification step, which facilitates the migration of electrons along the entire conjugated skeleton, thereby improving the photocatalytic activity. The porosity of the COFs was evaluated by N₂ adsorption measurements at 77 K, as depicted in Figure 2f. As shown in Figure S3b,c (Supporting Information), the ‐NH_2_ and ‐OH groups of PTTN‐AO were assigned to the N 1s (400.0 eV) and O 1s (532.2 eV) spectra, further affirming the successful hydrolysis of CN into AO.^[^ ^13^ ^]^

**Figure 3:**
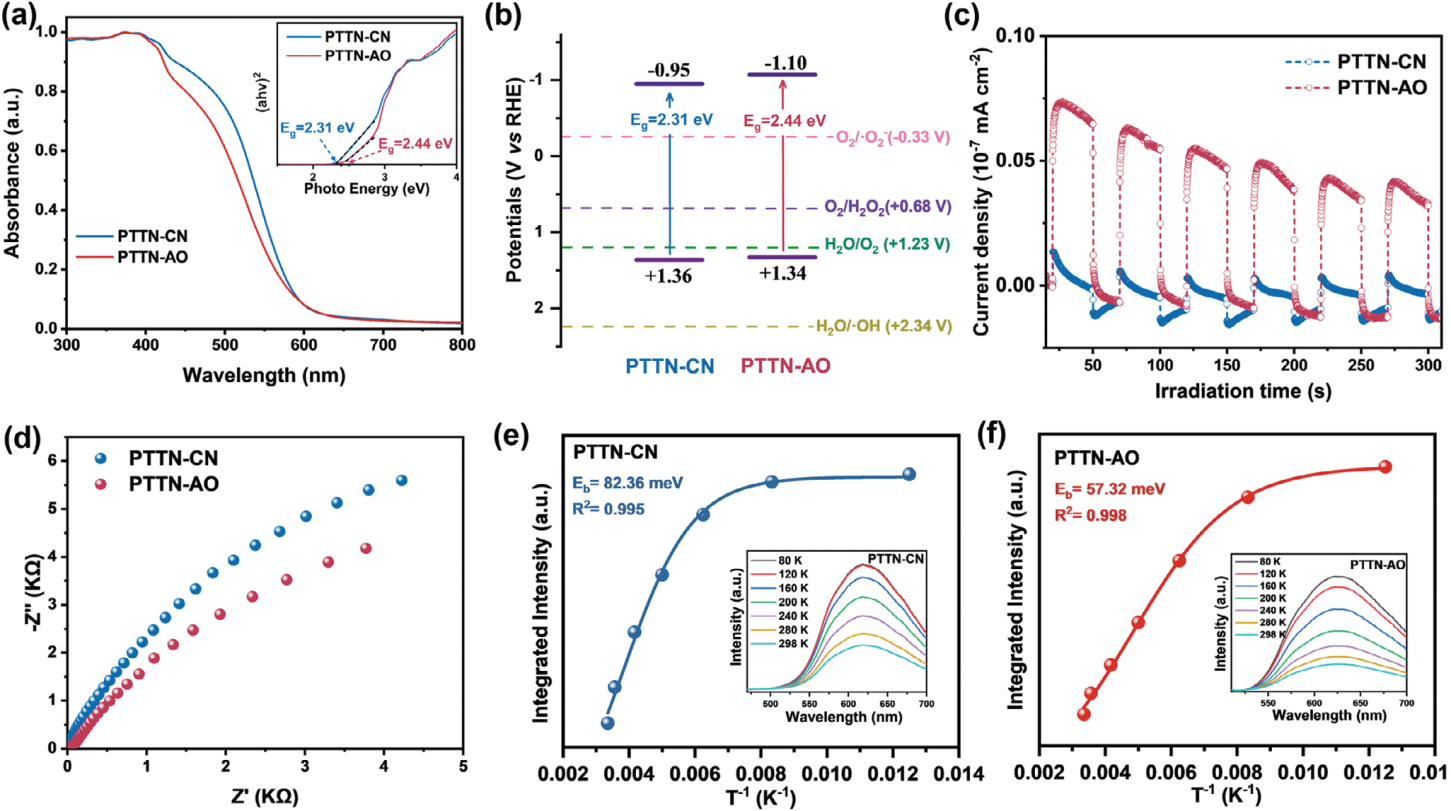
a) UV/vis absorption spectra and Tauc plot (inset); b) experimentally derived energy band alignments; c) transient photocurrent responses; d) EIS Nyquist plots; and e,f) integrated PL emission intensity as a function of temperature (inset: temperature‐dependent PL spectra from 80 to 298 K) of PTTN‐CN and PTTN‐AO.

The Brunauer‐Emmett‐Teller (BET) surface areas of PTTN‐CN and PTTN‐AO were calculated to be 608.12 and 386.80 m^2^ g^−1^, respectively. The non‐local density functional theory (NL‐DFT) cylinder pore model revealed the pore size distributions of both COFs, which yielded distinct distribution peaks at 2.34 and 2.00 nm (Figure 2g), respectively, indicating that both COFs possess mesoporous structures that facilitate the adsorption of O₂ and the diffusion of water, making them suitable for photocatalytic reduction to produce H₂O₂.^[^ ^14^ ^]^ Scanning electron microscopy (SEM) images showed that both PTTN‐CN and PTTN‐AO present rod‐like morphologies (Figures S5 and S6, Supporting Information), with small grafts observed on the rods of PTTN‐AO. High‐resolution transmission electron microscopy (HR‐TEM) disclosed the regular lattice fringes in both COFs, with lattice spacings of 2.30 and 2.00 nm, respectively, also validating their crystallinity (Figure 2h,i; Figure S7, Supporting Information).The UV–vis diffuse reflectance spectra (UV‐Vis DRS) of both COFs exhibited strong absorption in the region of 300–650 nm, indicating good visible light absorption (**Figure** **3**a). The corresponding Tauc plots derived from the UV–vis DRS determined the optical band gap (*E*_g_) of 2.31 and 2.44 eV, respectively (inset in Figure 3a). XPS valence band spectra (XPS‐VB), presented in Figure S8 (Supporting Information), were utilized to calculate their energy levels of the valence band (*E*_VB_), giving the values of +1.36 and +1.34 V for PTTN‐CN and PTTN‐AO relative to the Relative Hyedrogen Electrode (RHE), respectively. Consequently, the corresponding conduction band (*E*_CB_) were calculated based on the formula *E*_CB_ = *E*_VB_−*E*_g_, showing the values of −0.95 and ‐1.10 V (vs RHE), respectively. The energy diagram shown in Figure 3b, the potentials of all *E*_CB_ are more negative than the potentials for both the direct 2e^−^ ORR (+0.68 V vs RHE) and the stepwise 1e^−^ ORR (−0.33 V vs RHE), while the *E*_VB_ meet the potential requirement for the 4‐electron water oxidation reaction (4e^−^ WOR) to produce O₂.^[^ ^15^ ^]^ This indicates that both COFs are thermodynamically capable of driving overall photocatalytic H_2_O_2_ production without any sacrificial agents. Interestingly, PTTN‐AO not only inherits the excellent light capture performance of its parent structure (PTTN‐CN), but also significantly improves the charge‐separation kinetics. As demonstrated in transient photocurrent responses and electrochemical impedance curves (Figure 3c,d), PTTN‐AO exhibited higher photocurrent density and lower charge‐transfer resistance than that of PTTN‐CN, indicating its enhanced charge separation and transport capabilities. The exciton binding energy (*E*_b_), a key parameter determined exciton dissociation and charge separation, were calculated employing the Arrhenius expression *I(T)* = *I_0_ /* [1 + *A*exp(−*E_b_/k_B_T*)],^[^ ^16^ ^]^ and the corresponding values were 82.36 and 57.32 meV for PTTN‐CN and PTTN‐AO, respectively (Figure 3e,f). The *E*_b_ value of PTTN‐AO was significantly lower than that of PTTN‐CN, suggesting that PTTN‐AO undergoes internal exciton dissociation more easily and exhibits more rapid charge separation, which enhanced photocatalytic H₂O₂ production.

**Figure 4:**
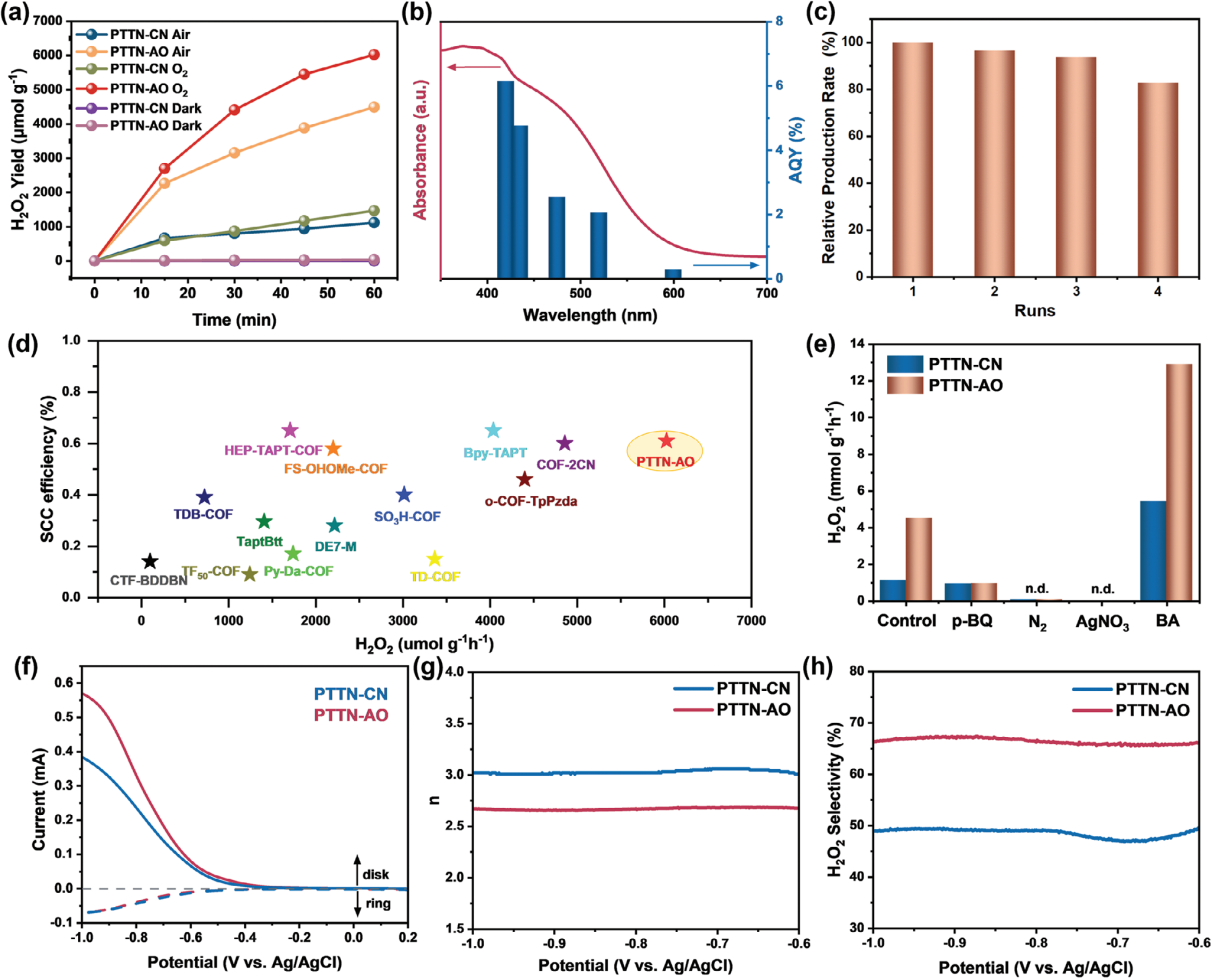
a) Photocatalytic H_2_O_2_ yield rates for PTTN‐CN and PTTN‐AO; b) UV/vis DRS spectrum and AQY comparison of PTTN‐AO; c) cycling performance of PTTN‐AO; d) summarized H_2_O_2_ production rate and SCC efficiencies; e) comparison of H_2_O_2_ production rates by PTTN‐CN and PTTN‐AO under different conditions; f) Linear Sweep Voltammetry (LSV) curves of PTTN‐CN and PTTN‐AO; g) the ORR electron transfer number calculated from RRDE measurement of in O_2_ pre‐saturated 0.1 m Na_2_SO_4_; h) H_2_O_2_ selectivity of PTTN‐CN and PTTN‐AO.

Then, as shown in **Figure** **4**a, under oxygen‐saturated conditions, the H₂O₂ production rates of PTTN‐AO and PTTN‐CN were 6024 and 1466 µmol h⁻¹g⁻¹, respectively, with the former being 4.1 times higher than the latter. Its activity was also superior to most previously reported COF‐based photocatalysts, showing that the AO sites played a critical role in enhancing the photocatalytic activity of PTTN‐AO. In consideration of the poor stability of H₂O₂, especially under light irradiation, the degradation of H₂O₂ by the COFs was also studied.^[^ ^17^ ^]^ As shown in Figure S9 (Supporting Information), the concentration of H₂O₂ in the aqueous solution remains almost unchanged, indicating that the difference in H₂O₂ production between the PTTN‐CN and PTTN‐AO was not due to decomposition of H₂O₂. Additionally, the highest H_2_O_2_ production rate of 32.1 µmol h⁻¹ was achieved with 10 mg of COF (Figure S10, Supporting Information).The apparent quantum yield (AQY) of PTTN‐AO was 6.15% at 420 nm, and the AQY decreased with increasing wavelength, following the trend of its visible light absorption intensity (Figure 4b).^[^ ^18^ ^]^ Moreover, under simulated sunlight (AM 1.5G), PTTN‐AO achieved a SCC efficiency of 0.61%, surpassing most previously reported COF‐based materials and making it one of the most efficient photocatalysts for H₂O₂ production to date (Figure 4d; Table S1, Supporting Information). Notably, PTTN‐AO also demonstrated high photocatalytic activity for H₂O₂ production in natural seawater (Figure S11, Supporting Information), underscoring its broad applicability. Impressively, as presented in Figure 4c, the H_2_O_2_ production rate of PTTN‐AO slightly decreased over four consecutive cycles, suggesting its outstanding photocatalytic recyclability. After multiple catalytic cycles, there was no significant difference in FT‐IR spectra and PXRD spectra, highlighting the structural integrity of PTTN‐AO during the photocatalytic process (Figure S12, Supporting Information). In addition, the sustained production H_2_O_2_ performance of PTTN‐AO was shown in Figure S13 (Supporting Information).^[^ ^19^ ^]^ The accumulated H_2_O_2_ concentrations were exceed 1 mmol L^−1^ after five hours irradiation, reaching the threshold required for sewage purification. For example, the typical organic pollutant Rhodamine B (RhB) was almost completely degraded via the Fenton reaction within 5 minutes (Figure S14, Supporting Information).^[^ ^20^ ^]^

A series of control experiments were conducted to further reveal the reaction route associated with the photocatalytic H₂O₂ generation. As shown in Figure 4e, almost no H₂O₂ was detected under N₂ or dark conditions, which indicates that the H₂O₂ formation pathway in our study is driven by photocatalytic ORR, rather than WOR.^[^ ^21^ ^]^ Quenching experiments were also conducted to identify the role of active species involved in redox process.^[^ ^22^ ^]^ Silver nitrate (AgNO_3_), benzyl alcohol (BA), and *p*‐benzoquinone (*p*‐BQ) were used as scavengers for electrons (e⁻), holes (h⁺), and superoxide radicals (·O₂⁻), respectively. Both COFs produced negligible H_2_O_2_ in the presence of AgNO_3_, confirming the electron‐driven nature of the ORR.^[^ ^23^ ^]^

In contrast, the introduction of BA significantly increased the H₂O₂ production rate of PTTN‐AO to 12.90 mmol h⁻¹g⁻¹, while PTTN‐CN reached 5.42 mmol h⁻¹g⁻¹, further verifying the electron‐mediated ORR mechanism. Furthermore, the addition of *p*‐BQ resulted in a markly decrease in H₂O₂ production for PTTN‐AO, which was ascribable to ·O₂⁻ quenching, indicating ·O₂⁻ was a key intermediate during the ORR process.^[^ ^24^ ^]^ Reasonably, PTTN‐AO primarily follows a two‐step 1e^−^ pathway (O₂ → ·O₂⁻ → H₂O₂), with some contribution from one‐step 2e^−^ pathway (O₂ + 2e⁻ + 2H⁺ → H₂O₂) for H₂O₂ production. For PTTN‐CN, the H₂O₂ yield slightly decreased upon the addition of *p*‐BQ, signaling that it primarily follows the one‐step 2e^−^ pathway, with minor contribution from the two‐step 1e^−^ pathway. Overall analysis, post‐functionalization through AO conversion can greatly enhance the efficiency of photocatalytic H₂O₂ production. During the photocatalytic process, O₂ can be reduced to H₂O₂ via a 2e⁻ ORR or reduced to water via a four‐electron (4e⁻) reaction (O₂ + 4e⁻ + 4H⁺ → 2H₂O).^[^ ^25^ ^]^ Electrochemical analysis using rotating ring‐disk electrode (RRDE) reveal that PTTN‐AO manifests a lower oxygen reduction water‐generation current and a higher 2e⁻ ORR H₂O₂‐generation current compared to PTTN‐CN (Figure 4f). The average electron transfer numbers for ORR on PTTN‐CN and PTTN‐AO are calculated to be 3.0 and 2.7, respectively (Figure 4g), further implying that PTTN‐AO participate in more 2e^−^ ORR events than PTTN‐CN. As shown in Figure 4h, PTTN‐AO exhibits a H₂O₂ selectivity of 66% in the potential range of ‐0.6 to ‐1.0 V, significantly higher than the ∼49% selectivity of PTTN‐CN. This distinct difference highlights the ability of PTTN‐AO to selectively promote H₂O₂ production, suggesting that PTTN‐AO is kinetically more favorable for the 2e⁻ ORR pathway.

**Figure 5:**
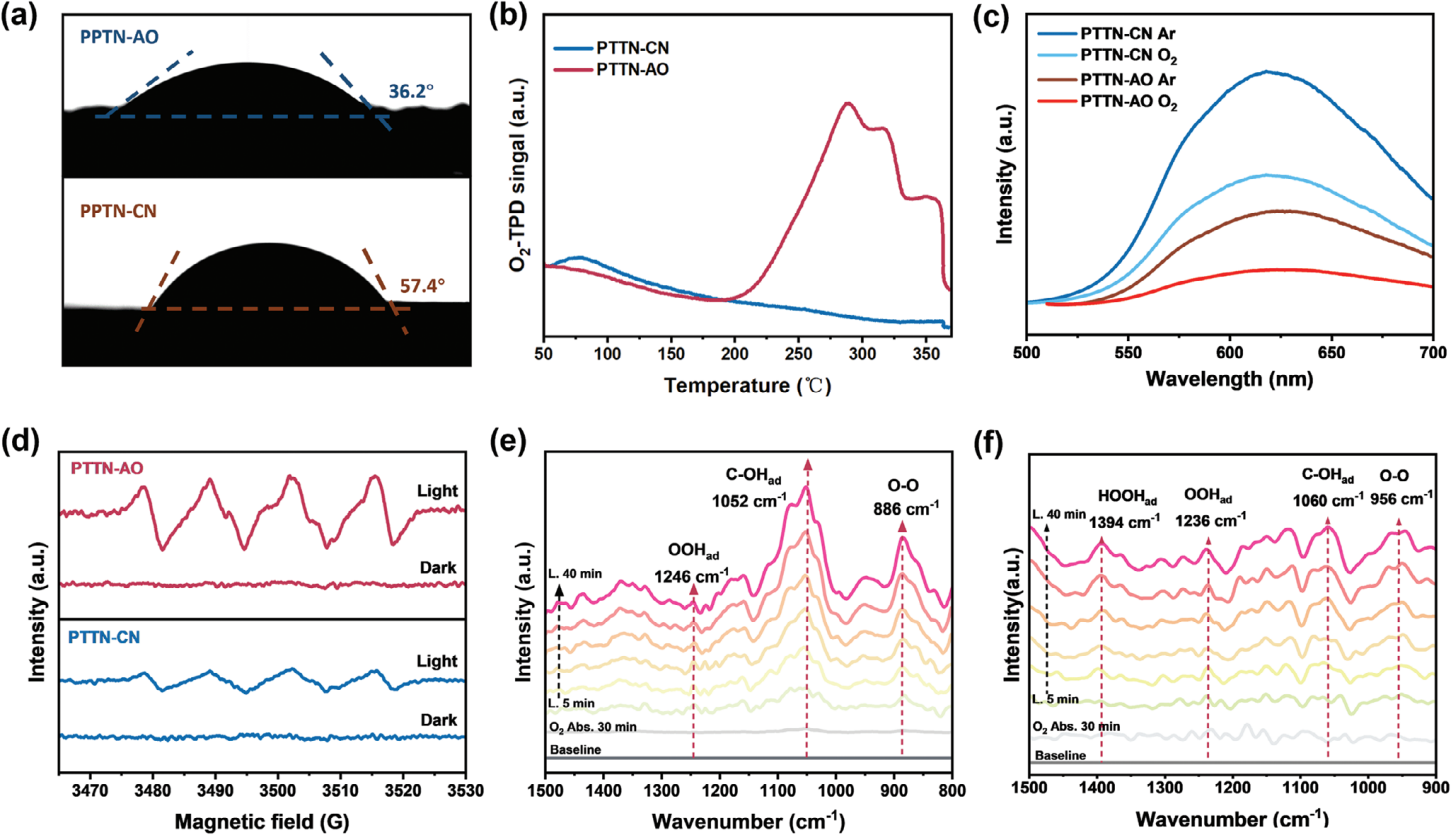
a) The differences in hydrophilicity for PTTN‐CN and PTTN‐AO using contact angle measurements; b) the O_2_‐TPD of PTTN‐CN and PTTN‐AO; c) fluorescence spectra of PTTN‐CN and PTTN‐AO under Ar and O_2_ atmospheres; d) EPR spectra of PTTN‐CN and PTTN‐AO in dark and light irradiation in DMPO under O_2_ atmosphere; in situ DRIFTS spectra of e) PTTN‐CN and f) PTTN‐AO.

To understand the mechanism of PTTN‐AO enhancing H₂O₂ production, we investigated the O₂ and water adsorption capacities of PTTN‐CN and PTTN‐AO, along with their surface hydrophilicity. As shown in **Figure** **5**a, the contact angle of PTTN‐CN and PTTN‐AO were 57.4° and 36.2°, respectively. The more hydrophilic surface of PTTN‐AO derives from the AO groups, which can accelerate proton extraction and transfer, as well as stabilize O₂ adsorption, thus facilitating the photocatalytic O₂‐to‐H₂O₂ conversion process.^[^ ^26^ ^]^ Oxygen temperature‐programmed desorption (O₂‐TPD) results showed that PTTN‐AO had a larger desorption peak and a higher desorption temperature (≈289 °C) than PTTN‐CN (Figure 5b), indicating that the introduction of AO sites augmented the chemisorption ability for O₂. As shown in Figure 5c, the PL quenching efficiency of PTTN‐AO was higher than that of PTTN‐CN under O₂ atmosphere, suggesting that the introduction of AO was beneficial for the transfer of photo‐generated electrons to O_2_.

A detailed investigation of intermediates involved in the 2e⁻ ORR, such as **·**O₂⁻, was conducted to gain beyond insight into the reaction pathway. Electron spin resonance (ESR), a highly effective technique for detecting reactive oxygen species (ROS), was used with 5,5‐dimethyl‐1‐pyrroline N‐oxide (DMPO) as the spin trap.^[^ ^27^ ^]^ No signals for **·**OH intermediates were detected for PTTN‐CN and PTTN‐AO under both dark and light conditions (Figure S15, Supporting Information). This is consistent with the results of undetected H₂O₂ under N₂ conditions, indicating that these two COFs do not produce H₂O₂ via the 2e^−^ WOR pathway. Additionally, as presented in Figure 5d, PTTN‐AO exhibited a distinct six‐line signal characteristic of DMPO‐**·**O₂⁻ adduct under light irradiation, while the corresponding signal in PTTN‐ CN was denoting that the introduction of the AO group markedly increased **·**O₂⁻ generation. Then, in situ diffuse reflectance infrared Fourier transform spectroscopy (DRIFTS) was employed to unveil the intermediate species formed during the photocatalytic process.^[^ ^28^ ^]^ As shown in Figure 5e,f, during the initial 30 min under saturated oxygen in the dark, both spectra displayed only weak vibrational peaks, indicating that the reaction did not proceed in the absence of light. Notably, PTTN‐CN displayed a characteristic O‐O stretching band at 886 cm⁻¹, corresponding to the formation of an internal peroxide intermediate in the direct 2e^−^ ORR pathway. As well, the 1246 cm⁻¹ *OOH signal confirmed the stepwise 1e^−^ ORR pathway for H₂O₂ production. Surprisingly, PTTN‐AO exhibited both an O‐O peak at 956 cm⁻¹ and an *OOH signal at 1236 cm⁻¹, providing strong evidence that AO‐functionalized COFs supported both the direct 2e⁻and the stepwise 1e⁻ORR pathways for H₂O₂ production. The peaks at 1052 (PTTN‐CN) and 1060 cm⁻¹ (PTTN‐AO) were associated with the intermediate states in the 4e⁻WOR for O₂ evolution. Therefore, the time‐dependent generation of O₂ for both COFs under excess AgNO_3_ conditions was achieved (Figure S16, Supporting Information). These in situ infrared results align well with both the EPR and RRDE experimental results.

**Figure 6:**
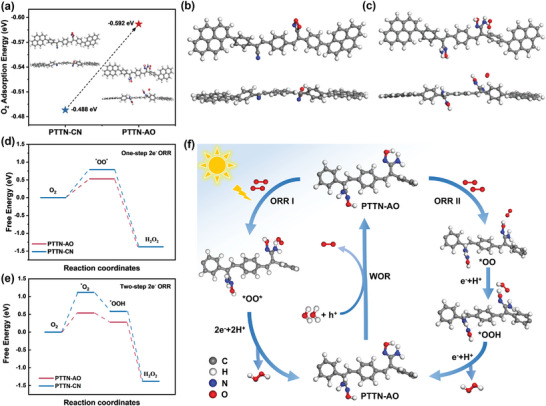
a) The adsorption energy of O_2_ for PTTN‐CN and PTTN‐AO; the O_2_ adsorption site and configuration on b) PTTN‐CN and c) PTTN‐AO; d) free‐energy diagrams for one‐step 2e^−^ ORR to H_2_O_2_ on the PTTN‐CN and PTTN‐AO; e) free‐energy diagrams for two‐step 1e^−^ ORR to H_2_O_2_ on the PTTN‐CN and PTTN‐AO; f) proposed reaction mechanism toward H_2_O_2_ production on the PTTN‐AO.

Accordingly, density functional theory (DFT) calculations were performed to investigate O₂ adsorption and the changes in free energy on both COFs during photocatalytic process. Optimized adsorption configurations for O₂ at different sites showed that the CN sites in PTTN‐CN featured stronger O₂ adsorption energies (**Figure** **6**b; Figure S17, Supporting Information). However, after introducing the AO groups, PTTN‐AO displayed a greater preference for O₂ adsorption at the AO sites (Figure 6c; Figure S18, Supporting Information). Moreover, the optimal O₂ adsorption energy for PTTN‐AO (‐0.592 eV) was substantially higher than that of PTTN‐CN (‐0.488 eV) (Figure 6a). Enhanced O₂ adsorption affinity was attributed to the hydrogen bonding facilitated by the AO groups, stabilizing the adsorbed O₂, in agreement with the O₂‐TPD experimental results. On the one hand, O₂ prefers to adsorb onto the CN site in PTTN‐CN, forming a C‐O‐O‐N internal peroxide species (O₂ + 2e⁻ → *OO*), which then desorbs as H₂O₂ via the 2e⁻ and 2H⁺ pathway. On the other hand, in PTTN‐AO, O₂ is simultaneously adsorbed by the ‐OH, ‐NH₂, and C = N bonds within the AO groups, and subsequently generates H_2_O_2_ through a direct 2‐electron reaction.

As shown in Figure 6d, the Gibbs free energy of the rate determining step (O₂ + 2e⁻ → *OO*) of direct 2e^−^ ORR for PTTN‐AO (0.53 eV) was 0.26 eV smaller than that of PTTN‐CN (0.79 eV), indicating that the AO‐functionalization strategy promoted the direct 2e^−^ ORR pathway for photocatalytic H_2_O_2_ production. Furthermore, the cyanide‐linked carbon atoms in PTTN‐CN and the OH groups in PTTN‐AO can also adsorb O₂ to form *OO, which desorbs as *OOH (O₂* + H⁺ + e⁻ → *OOH), then the *OOH receives an additional electron and proton to break the ‐OOH bond, forming H₂O₂ (*OOH + e⁻ + H⁺ → H₂O₂). As shown in Figure 6e and Figure S19 (Supporting Information), the Gibbs free energies of the rate‐limiting step (O₂* + H⁺ + e⁻ → *OOH) for PTTN‐AO (0.54 eV) was 0.58 eV lower than that of PTTN‐CN (1.12 eV), further suggesting that the AO‐functionalization strategy also encouraged the two‐step 1e^−^ ORR pathway for photocatalytic H_2_O_2_ production. These results comprehensively demonstrated that AO functionalization strategy enhanced the activation of O_2_ and reduced the energy barrier of both one‐step 2e^−^ and two‐step 1e^−^ ORR pathways, thus increasing the photocatalytic H_2_O_2_ production for PTTN‐AO. The corresponding possible mechanism diagram of PTTN‐AO and PTTN‐CN has been drawn in Figure 6f and Figure S20 (Supporting Information). The PTTN‐AO adsorbs O₂ from the environment via AO sites, which then reacts with photo‐generated electrons and protons through two distinct ORR pathways (ORR I and ORR II) to generate H₂O₂. Simultaneously, photo‐generated holes drive the 4e⁻ WOR process with water to produce O₂, thereby sustaining the catalytic cycle necessary for continuous H₂O₂ production.

## 3 Conclusion

In summary, we have successfully achieved efficient photocatalytic H₂O₂ production from pure water and oxygen via a 2e^−^ ORR by a universal post‐functionalization strategy that convert the CN groups to AO groups in COFs. As revealed by experimental studies, the AO groups played a key role in improving exciton dissociation and charge transfer, enhancing surface hydrophilicity, promoting proton transfer, as well as increasing the oxygen adsorption capacity, which was crucial for collectively promoting the photosynthesis of H_2_O_2_. Further DFT calculations suggested that the introduction of AO groups simultaneously reduced the energy barrier of both one‐step 2e^−^ and two‐step 1e^−^ ORR pathways for generation of H_2_O_2_, which promoted the photocatalytic activity of PTTN‐AO. As a result, PTTN‐AO achieved a superior H₂O₂ production rate of 6024 µmol h⁻¹g⁻¹ in pure water, with a high SCC value 0.61%. Our research offers a general and green post‐modification method to develop the metal‐free COF‐based photocatalysts for efficient O_2_‐to‐H_2_O_2_ conversion, and enrich the modification toolboxes of COFs.

## Conflict of Interest

The authors declare no conflict of interest.

## Supporting information



Supporting Information

## Data Availability

The data that support the findings of this study are openly available in public repository at [https://doi.org/10.1002/advs.202415194].

## References

[1] a) R. Hage , A. Lienke , Angew. Chem., Int. Ed. 2005, 45, 206; 10.1002/anie.200500525 16342123

[2] a) I. Yamanaka , T. Murayama , Angew. Chem., Int. Ed. 2008, 47, 1900; 10.1002/anie.200704431 18224637

[3] a) Y. Kofuji , Y. Isobe , Y. Shiraishi , H. Sakamoto , S. Tanaka , S. Ichikawa , T. Hirai , J. Am. Chem. Soc. 2016, 138, 10019; 27439985 10.1021/jacs.6b05806

[4] a) Z. Teng , Q. Zhang , H. Yang , K. Kato , W. Yang , Y.‐R. Lu , S. Liu , C. Wang , A. Yamakata , C. Su , B. Liu , T. Ohno , Nat. Catal. 2021, 4, 374;

[5] a) H. Yu , F. Zhang , Q. Chen , P. K. Zhou , W. Xing , S. Wang , G. Zhang , Y. Jiang , X. Chen , Angew. Chem., Int. Ed. 2024, 63, e202402297; 10.1002/anie.202402297 38488772

[6] a) A. P. Côté , A. I. Benin , N. W. Ockwig , M. O'Keeffe , A. J. Matzger , O. M. Yaghi , Science 2005, 310, 1166; 16293756 10.1126/science.1120411

[7] a) Y. Yusran , X. Guan , H. Li , Q. Fang , S. Qiu , Natl. Sci. Rev. 2020, 7, 170; 34692030 10.1093/nsr/nwz122 PMC8288834

[8] a) J. Xu , C. Yang , S. Bi , W. Wang , Y. He , D. Wu , Q. Liang , X. Wang , F. Zhang , Angew. Chem., Int. Ed. 2020, 59, 23845; 10.1002/anie.202011852 32954597

[9] X. Di , X. Lv , H. Wang , F. Chen , S. Wang , G. Zheng , B. Wang , Q. Han , Chem. Eng. J. 2023, 455,140124.

[10] Y. Hou , P. Zhou , F. Liu , Y. Lu , H. Tan , Z. Li , M. Tong , J. Ni , Angew. Chem., Int. Ed. 2024, 63, e202318562. 10.1002/anie.202318562 38151472

[11] B. Ahmed , Z. Ahmad , A. Khatoon , I. Khan , N. Shaheen , A. A. Malik , Z. Hussain , M. A. Khan , Environ. Sci. Pollut. Res. 2023, 30, 103496. 10.1007/s11356-023-29589-0 37704807

[12] B. Zhang , M. Wei , H. Mao , X. Pei , S. A. Alshmimri , J. A. Reimer , O. M. Yaghi , J. Am. Chem. Soc. 2018, 140, 12715. 30247881 10.1021/jacs.8b08374

[13] Y. Wang , Y. Z. Cheng , K. M. Wu , D. H. Yang , X. F. Liu , X. Ding , B. Han , Angew. Chem., Int. Ed. 2023, 62, e202310794. 10.1002/anie.202310794 37596246

[14] Y. Xu , S. Jin , H. Xu , A. Nagai , D. Jiang , Chem. Soc. Rev. 2013, 42, 8012. 23846024 10.1039/c3cs60160a

[15] A. Alam , B. Kumbhakar , A. Chakraborty , B. Mishra , S. Ghosh , A. Thomas , P. Pachfule , ACS Mater. Lett. 2024, 6, 2007. 10.1002/adma.202413118 PMC12747479 39654345

[16] a) M. Rahman , H. Tian , T. Edvinsson , Angew. Chem., Int. Ed. 2020, 59, 16278; 10.1002/anie.202002561 PMC7540687 32329950

[17] a) T. Xu , Z. Wang , W. Zhang , S. An , L. Wei , S. Guo , Y. Huang , S. Jiang , M. Zhu , Y. B. Zhang , W. Zhu , J. Am. Chem. Soc. 2024, 146, 20107; 38842422 10.1021/jacs.4c04244

[18] J. N. Chang , J. W. Shi , Q. Li , S. Li , Y. R. Wang , Y. Chen , F. Yu , S. L. Li , Y. Lan , Angew. Chem., Int. Ed. 2023, 62, e202303606. 10.1002/anie.202303606 37277319

[19] Q. Liao , Q. Sun , H. Xu , Y. Wang , Y. Xu , Z. Li , J. Hu , D. Wang , H. Li , K. Xi , Angew. Chem., Int. Ed. 2023, 62, e202310556. 10.1002/anie.202310556 37632257

[20] Z. Zhang , Q. Zhang , Y. Hou , J. Li , S. Zhu , H. Xia , H. Yue , X. Liu , Angew. Chem., Int. Ed. 2024, 63, e202411546. 10.1002/anie.202411546 38949611

[21] J. Y. Yue , J. X. Luo , Z. X. Pan , Q. Xu , P. Yang , B. Tang , Angew. Chem., Int. Ed. 2024, 137, e202417115. 10.1002/anie.202417115 39363753

[22] S. Feng , H. Cheng , F. Chen , X. Liu , Z. Wang , H. Xu , J. Hua , ACS Catal. 2024, 14, 7736.

[23] S. Feng , L. Wang , L. Tian , Y. Liu , K. Hu , H. Xu , H. Wang , J. Hua , Chem. Sci. 2024, 15, 11972. 39092094 10.1039/d4sc02832e PMC11290433

[24] R. Liu , Y. Chen , H. Yu , M. Položij , Y. Guo , T. C. Sum , T. Heine , D. Jiang , Nat. Catal. 2024, 7, 195.

[25] H. Cheng , J. Cheng , L. Wang , H. Xu , Chem. Mater. 2022, 34, 4259.

[26] L. Li , X. Lv , Y. Xue , H. Shao , G. Zheng , Q. Han , Angew. Chem., Int. Ed. 2024, 63, e202320218. 10.1002/anie.202320218 38353181

[27] S. Wang , Z. Xie , D. Zhu , S. Fu , Y. Wu , H. Yu , C. Lu , P. Zhou , M. Bonn , H. I. Wang , Q. Liao , H. Xu , X. Chen , C. Gu , Nat. Commun. 2023, 14, 6891. 37898686 10.1038/s41467-023-42720-6 PMC10613291

[28] T. Yang , D. Zhang , A. Kong , Y. Zou , L. Yuan , C. Liu , S. Luo , G. Wei , C. Yu , Angew. Chem., Int. Ed. 2024, 63, e202404077. 10.1002/anie.202404077 38494453

