# Supplementary material for: Amidoxime‐Functionalized sp 2‐Carbon‐Conjugated Covalent Organic Frameworks for Overall Photocatalytic Hydrogen Peroxide Production
Source: Adv Sci (Weinh). 2025 Feb 18;12(14):2415194. doi: 10.1002/advs.202415194 (PMC11984853; doi:10.1002/advs.202415194)
Supplement: Supplementary file 1 — Supporting Information [file ADVS-12-2415194-s001.docx]

**Supporting Information**

**Amidoxime-Functionalized *sp^2^*-Carbon-Conjugated Covalent Organic Frameworks for** **Overall Photocatalytic Hydrogen Peroxide Production**

Zhiwu Yu, ^[a, †]^ Fengtao Yu, ^*[b, †]^ Mei Xu, ^[b]^ Shufan Feng, ^[a]^ Jianding Qiu, ^*[b]^ Jianli Hua*^[a]^
*^a^ Key Laboratory for Advanced Materials and Joint International Research Laboratory for Precision Chemistry and Molecular Engineering, Feringa Nobel Prize Scientist Joint Research Center, Frontiers Science Center for Materiobiology and Dynamic Chemistry, School of Chemistry and Molecular Engineering, East China University of Science and Technology Shanghai, 200237, China
E-mail:* [*jlhua@ecust.edu.cn*](mailto:jlhua@ecust.edu.cn)

*^b^ State Key Laboratory of Nuclear Resources and Environment, East China University of Technology, Nanchang, 330013, People’s Republic of China*

*E-mail: fty853815622@ecut.edu.cn; jdqiu@ecut.edu.cn*

*^†^ These authors contributed equally to this work.*

Table of Contents

[**Section S1. Materials and Methods 4**](#_Toc187314388)

[**S1.1 Materials 4**](#_Toc187314389)

[**S1.2 Instruments 4**](#_Toc187314390)

[**S1.3 Photocatalytic H_2_O_2_ measurements 4**](#_Toc187314391)

[**S1.4 Quenching experiments measurements 5**](#_Toc187314392)

[**S1.5 Apparent quantum yield (AQY) measurement 5**](#_Toc187314393)

[**S1.6 SCC efficiency measurements 5**](#_Toc187314394)

[**S1.7 Electrochemical rotating ring-disk electrode (RRDE) measurement 5**](#_Toc187314395)

[**S1.8 Oxygen temperature programed desorption (O_2_-TPD) 6**](#_Toc187314396)

[**S1.9 In situ diffuse reflectance infrared fourier transform spectroscopy (DRIFTS) 6**](#_Toc187314397)

[**S1.10 DFT theoretical calculations method 6**](#_Toc187314398)

[**S1.11 RhB degradation 7**](#_Toc187314399)

[**S1.12 Reactor setup photograph 7**](#_Toc187314400)

[**S1.12 Synthesis of Py-CN 9**](#_Toc187314401)

[**S1.13 Synthesis of PTTN-CN COF 9**](#_Toc187314402)

[**S1.14 Synthesis of PTTN-AO COF 10**](#_Toc187314403)

[**Section S2. Characterization Figures 11**](#_Toc187314404)

[**Figure S1. 11**](#_Toc187314405)

[**Figure S2. 11**](#_Toc187314406)

[**Figure S3 11**](#_Toc187314407)

[**Figure S4 12**](#_Toc187314408)

[**Figure S5. 12**](#_Toc187314409)

[**Figure S6. 13**](#_Toc187314410)

[**Figure S7 13**](#_Toc187314411)

[**Figure S8. 14**](#_Toc187314412)

[**Figure S9. 14**](#_Toc187314413)

[**Figure S10 15**](#_Toc187314414)

[**Figure S11 15**](#_Toc187314415)

[**Figure S12 16**](#_Toc187314416)

[**Figure S13 16**](#_Toc187314417)

[**Figure S14 17**](#_Toc187314418)

[**Figure S15 17**](#_Toc187314419)

[**Figure S16 18**](#_Toc187314420)

[**Figure S17 18**](#_Toc187314421)

[**Figure S18 19**](#_Toc187314422)

[**Figure S19 19**](#_Toc187314423)

[**Section S3. Supplementary Tables 20**](#_Toc187314424)

[**Table S1. Comparison of photocatalytic performances among recently reported COFs 20**](#_Toc187314425)

[**Table S2. Fractional atomic coordinates and the unit cell of PTTN-CN 22**](#_Toc187314426)

[**Section S4. Supporting References 25**](#_Toc187314427)

**Section S1. Materials and Methods**

**S1.1 Materials**

All starting materials and solvents, including Potassium carbonate (K_2_CO_3_), hydrochloric acid (HCl), dichloromethane (DCM), ethanol, tetrahydrofuran (THF), acetone, methanol (MeOH), benzyl alcohol ,1,4-Dioxane, dichlorobenzene (*o*-DCB), potassium hydroxide (KOH), 1,3,6,8-Tetrabromopyrene (Py-Br), 1,4-Benzenedicarboxaldehyde, triethylamine ,hydroxyl ammonium hydrochl-oride, 4-(cyanomethyl)benzeneboronic acid and Bis(triphenylphosphine)-palladium(II) chloride (PdCl_2_(PPh_3_)_2_) were acquired from TENSUS BIOTECH and Aladdin Chemicals. The purity of all purchased reagents and solvents was at least 95%, and they were used as received without further purification.

**S1.2 Instruments**

PXRD data were collected on a Bruker D8 Advance Powder X-ray Diffractometer using a Cu Kα source (λ = 1.5418 Å) over the range of 2*θ* = 2.0−30.0° with a step size of 0.02° and 2 s per step. Solid-state ^13^C-NMR spectra were acquired with the Bruker Advance III 500 MHz spectrometer. Fourier transform infrared (FT-IR) spectra were recorded using KBr pellets on a Nicolet Impact 410 spectrometer. The sample morphology was examined using both a field emission scanning electron microscope (FESEM, JSM-6360LV) and a transmission electron microscope (TEM, JEOL JEM-2100). X-ray photoelectron spectroscopy (XPS) analysis was performed with an ESCALAB 250Xi Thermo Scientific TM XPS equipment. Nitrogen adsorption and desorption at 77 K were carried out using a Micromeritics ASAP 2020 instrument, with the samples being degassed at 120 °C for 12 hours under a vacuum of 10^-5^ bar prior to analysis. The specific surface areas were calculated using the Brunauer-Emmett-Teller (BET) method, and the pore size distribution was determined from the sorption curve using the non-local density functional theory (NLDFT) model. Diffuse reflectance spectroscopy (DRS) measurements were performed with a Varian Cary 500 spectrophotometer. Electron spin resonance (ESR) spectra were recorded using the Bruker EMX nano instrument. All electrochemical tests were conducted in a standard three-electrode cell using a CHI760E S3S4 electrochemical workstation. Photoluminescence (PL) spectra were collected using a Hitachi F-4500 fluorescence spectrophotometer.

**S1.3 Photocatalytic H_2_O_2_ measurements**

5 mg photocatalyst was dispersed in 50 mL of solution. Subsequently, the dispersion underwent ultrasonic treatment for 10 minutes, followed by continuous stirring for 30 minutes. All photocatalytic reactions were conducted in an air atmosphere under the illumination of a 300 W Xenon Lamp, with a UV cut-off filter at 420 nm. At 15-minute intervals, 3 mL of the solution was sampled. To this solution, 1 mL of 0.1 mol∙L**^−1^** aqueous potassium hydrogen phthalate (C_8_H_5_KO_4_) solution and 1 mL of 0.4 mol∙L**^−1^** aqueous potassium iodide (KI) solution was added, and the mixture was allowed to sit for 3 hours. Under acidic conditions (H_2_O_2_ + 3I^−^ + 2H^+^→ I_3_^−^ + 2H_2_O), H_2_O_2_ molecules reacted with iodide anions (I^−^) to produce triiodide anions (I**_3_**^−^) with strong absorption around 350 nm. The quantity of I_3_^−^ was determined via UV–vis spectroscopy based on the absorbance at 350 nm, which allowed the estimation of the amount of H_2_O_2_ produced during each reaction (see below Figure S1a and S1b).


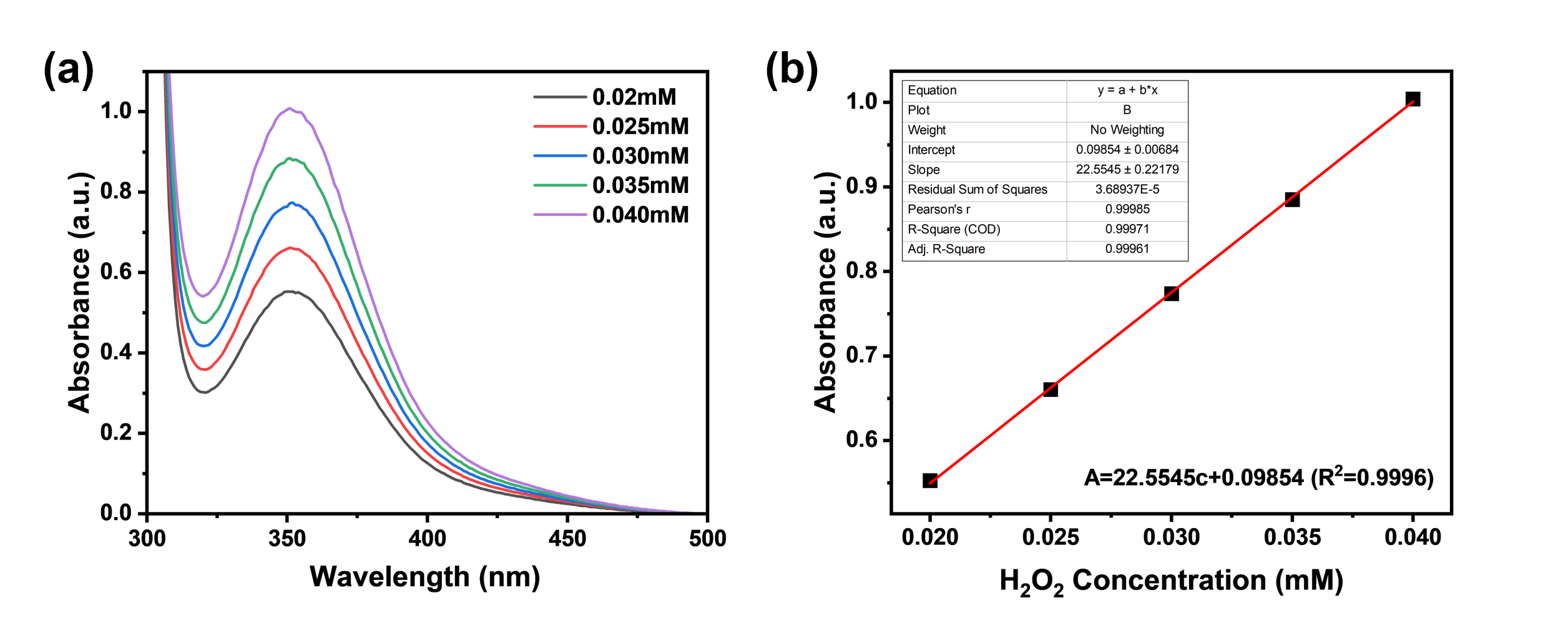


**Figure S1**. (a, b) the standard curve used for determining H₂O₂ concentration via the iodometric method.

S1.4 Quenching experiments measurements

5 mg photocatalyst was dispersed in 50 mL of solution. Subsequently, 100 mg of excess silver nitrate was added to the dispersion as an electron sacrificial agent; 100 mg of excess p-benzoquinone was added to the dispersion as a superoxide radical scavenger; 10% BA was used as a proton (H⁺) scavenger. The dispersion underwent ultrasonic treatment for 10 minutes, followed by continuous stirring for 30 minutes.

**S1.5 Apparent quantum yield (AQY) measurement**

The AQY of H_2_O_2_ over COF PTTN-AO was measured with irradiation light through different wavelength bandpass filter (420, 435, 475,520 and 600 nm). The photon flux of incident light was measured by a PL.MW200 photoradiometer (Beijing Perfectlight Co., Ltd.). The AQY was calculated according to the following equation:

$$AQY(\%)=\frac{2\times Number of evolved H_{2}O_{2}\mathrm{molecules}}{Number of incident photons}\times100\%=\frac{2\times C\times N_{A}}{S\times P\times\frac{\lambda}{h\times c}}\times100\%$$

Where *C* is the H_2_O_2_ production amount (μmol) per hour; *N_A_* is the Avogadro constant (6.02 × 10^23^ mol**^−1^**); *S* is the irradiation area (4 cm**^2^** ); *P* is the monochromatic light intensity (W cm**^−2^**) (*P* is detected by optical power meter); *t* is the light irradiation time (3600 s); *λ* is the wavelength of the monochromatic light (nm); *h* is the Plank constant (6.626 × 10^-34^ J s); *c* is the speed of light (3 × 10^8^ m s**^−1^**).

**S1.6 SCC efficiency measurements**

The solar-to-chemical energy conversion (SCC) efficiency was determined by using an AM 1.5 G solar simulator as the light source (300 W Xe lamp). The photocatalytic reaction was carried out in pure deionized water (10 mL) with photocatalysts (50 mg). Ultrasonication and O_2_ bubbling for half an hour respectively. During the photocatalytic tests, O₂ was continuously bubbled into the reaction vessel. The solar-to-chemical conversion (SCC) efficiency was calculated using the following equation:

$$SCC efficiency (\%)=\frac{[\Delta G forH_{2}O_{2}generation (Jmol ―1 )][H_{2}O_{2} formed (mol)]}{[Total input power(W)][Reaction time (s)]}\times100\%$$

Where ${\Delta G}_{H_{2}O_{2}}$= 117 kJ mol**^-1^** is the free energy for H_2_O_2_ generation (117 kJ mol**^−1^**). The overall irradiation intensity of the AM 1.5 global spectrum (300–2500 nm) is 1000 W m**^−2^** and the irradiation areas are 4 × 10**^−4^** m**^2^**.

**S1.7 Electrochemical rotating ring-disk electrode (RRDE) measurement**

The rotating ring disk electrode (RRDE, PINE Research Instrumentation, electrode area: 0.2475 cm^2^) was employed as the substrate for the working electrode. The preparation of the working electrode involves first weighing 10 mg of the sample and adding it to a mixture of 0.5 mL ethanol and 20 μL of 5 wt% Nafion solution. The mixture is sonicated for 10 minutes to ensure uniform dispersion. Then, 20 μL of the resulting ink is dropped onto a clean disk electrode, repeated twice, and allowed to air dry naturally before testing. The ORR catalytic test is then conducted using a rotating disk electrode as the substrate for the working electrode. A carbon rod serves as the counter electrode, and an Ag/AgCl electrode is used as the reference electrode. The potential is scanned from -1.0 to 0.2 V (vs. Ag/AgCl). The working electrode is placed in an oxygen-saturated 0.1 M phosphate-buffered saline (PBS, pH = 7) solution, and linear sweep voltammograms (LSV) is performed at a scan rate of 10 mV/s until the curve stabilizes at 1600 rpm. The transfer number electron (n) was calculated by the disk current (*I_d_*) and ring current (*I_r_*) results as the following equations:

$$n=4 \times\frac{I_{d}}{I_{d}+I_{r}/N}$$

$$H_{2}O_{2}\% = 200 \times\frac{I_{r}/N}{I_{d}+I_{r}/N}$$

Where *I_d_* and *I_r_* are the disk and ring current (mA), respectively, *N* is the collection

efficiency of the Pt ring (*N* = 0.37).

**S1.8 Oxygen temperature programed desorption (O_2_-TPD)**

O_2_-TPD was measured on Micromeritics AutoChem II 2920. The sample was pretreated in He flow under 300 °C for 1 h, and a pulse of 5% O_2_ in He was used for absorption of the oxygen molecules on the sample, followed by 1 h He flow with flow rate of 50 mL min^−1^ at 50 °C for removing the physically adsorbed oxygen molecules. O_2_-TPD was measured in the He flow with rate of 50 mL min^−1^, the initial temperature was 50 °C, and the ramp rate was 10 °C min^−1^. Desorbed oxygen was monitored by thermal conductivity detector (TCD).

**S1.9 In situ diffuse reflectance infrared fourier transform spectroscopy**

In situ diffuse reflectance infrared Fourier transform spectroscopy (DRIFTS) measurements were performed using a Bruker IFS 66v Fourier-transform spectrometer equipped with a Harrick diffuse reflectance accessory at the Infrared Spectroscopy and Microspectroscopy Endstation (BL01B). After Ar gas sweeps the sample for 30 min, vapor and O_2_ were purged into the reactor with the sample in the dark, and the container was closed after the ventilation was stopped. Ar was purged and sweeps the sample for 30 min, after which 300W xenon lamp (> 420nm) was used to illuminate the sample with vapor and Ar purging.

**S1.10 DFT theoretical calculations method**

In this study, all spin-polarized first-principles calculations were performed using the Vienna Ab-initio Simulation Package (VASP) developed by the University of Vienna for electronic structure calculations and quantum mechanical simulations.^[1]^ The Projector Augmented Wave (PAW) pseudopotential was employed to describe the interaction between electrons and ions, significantly reducing the computational load.^[2]^ Additionally, the exchange-correlation functional was treated using the Perdew-Burke-Enzerhof (PBE) functional within the framework of Generalized Gradient Approximation (GGA).^[3]^ The cutoff energy for the plane-wave basis set was set to 400 eV during the calculations. For structural relaxations, all atoms were allowed to optimize, with the k-point grid set to 2×2×1. The structure optimization was performed using the conjugate gradient method, with a force convergence criterion of 0.02 eV/Å. The self-consistent energy convergence criterion was set to 1×10⁻⁵ eV/atom.

The Gibbs free energy (G) of the system was defined as shown in Equation:

*∆*G = *∆*E_DFT + E_ZPE - T *∆*S

Where *∆*E_DFT represents the static electronic energy at 0 K, while E_ZPE, T, and *∆*S denote the zero-point energy, temperature, and entropy of the reaction, respectively. All the above VASP calculations were greatly accelerated using the "Quickly use Vienna Ab-initio Simulation Package" (qvasp) and VASPKIT software packages, which significantly enhanced computational efficiency.^[4]^

**S1.11 RhB degradation**

The 10 mg L^−1^ Rhodamine B (RhB) containing 0.1 M FeSO_4_·7H_2_O was prepared first. After that, taking out 1 mL above RhB solution into a 3 mL colorimetric tube and then 2 mL photocatalytic H_2_O_2_ solution (20 mg PTTN-AO in 50 mL pure water, λ > 420 nm Xe lamp) was gradually added. The color change of RhB and absorbance were recorded.

**S1.12 Reactor setup photograph**

The photographs of reactor setup and reaction solution for photocatalytic H_2_O_2_ generation were showed below.


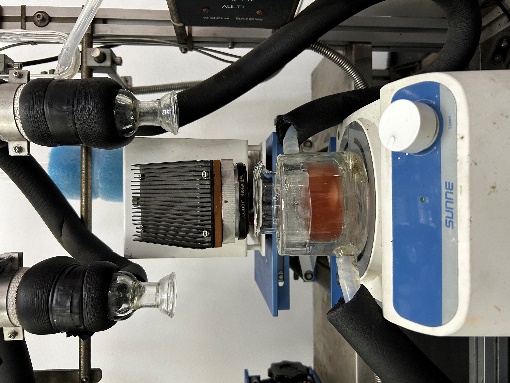

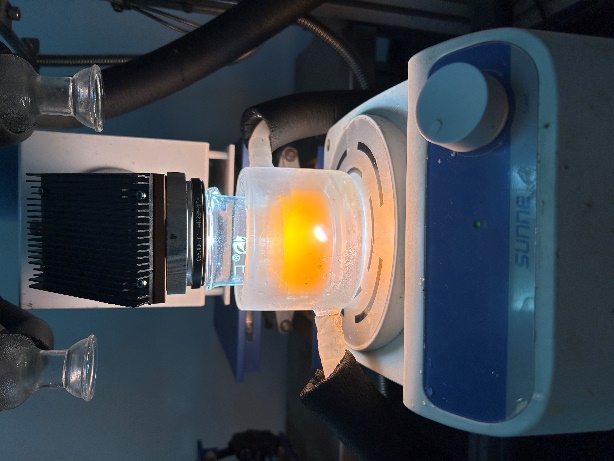


**S1.13 Synthesis of 2,2',2'',2'''-(Pyrene-1,3,6,8-tetrayltetrakis-(benzene-4,1-diyl))tetra-acetonitrile (Py-CN)**


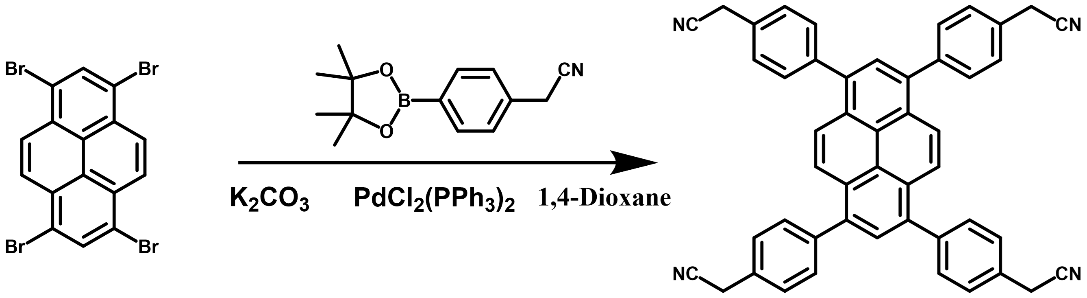


Based on a procedure reported in the literature.^[5]^ Py-Br (517.8 mg, 1.0 mmol), 4-(cyanomethyl)benzeneboronic acid (965.8 mg, 6.0 mmol), PdCl_2_(PPh_3_)_2_ (59.7 mg, 0.085 mmol), and K_2_CO_3_ (1.1 g, 8.0 mmol) were put into a two-necked flask, and the reaction system was subsequently vacuumed for 5 min, and protected with a nitrogen flow. The above process was repeated three times. 1,4-Dioxane (15 mL) was injected into the two-necked flask, and the mixture was heated to 85 °C and stirred for 3 days. After cooling to room temperature, the reaction mixture was washed with dilute hydrochloric acid (HCl/H_2_O = 1/6) and extracted with DCM. The combined organic layer was evaporated under reduced pressure. The crude product was purified by silica gel chromatography with DCM as the eluent to afford Py-CN as a light-yellow solid (245.1 mg, yield 37%).

**S1.14 Synthesis of PTTN-CN COF**


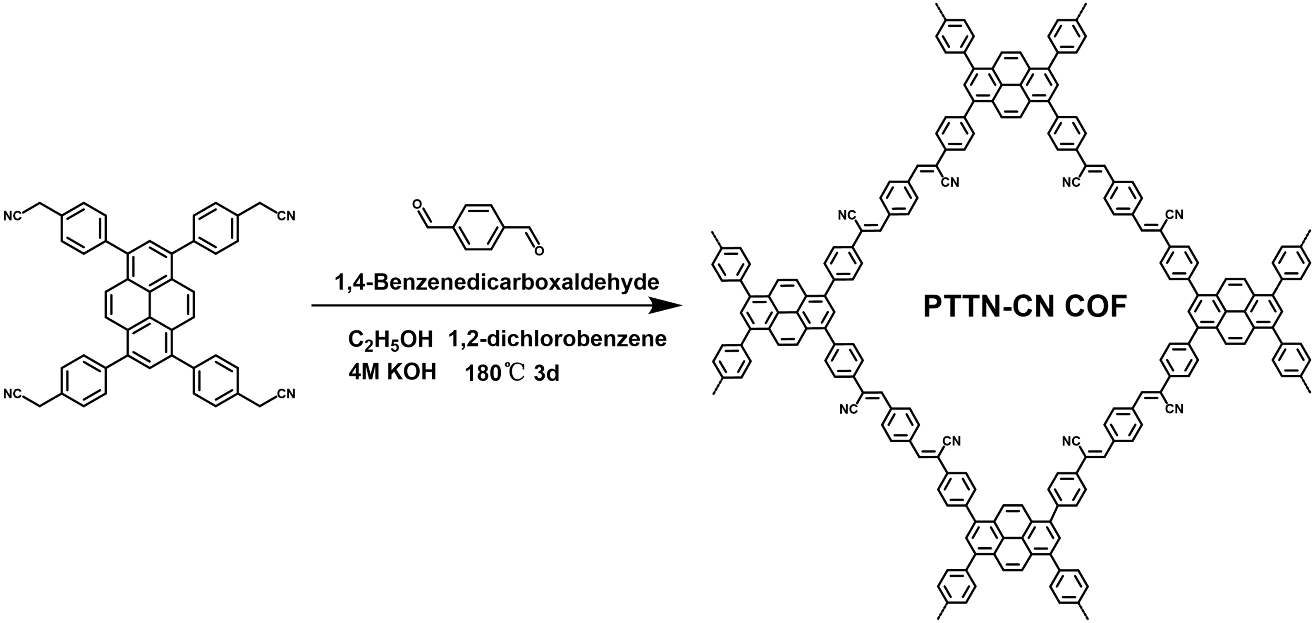


A Pyrex tube (10 mL) was charged with Py-CN (15.9 mg, 24.0 µmol), 1,4-benzenedicarboxaldehyde (6.5 mg, 48.2 µmol), ethanol (0.5 mL), 1,2-dichlorobenzene (0.5 mL), and aqueous KOH solution (4 M, 0.1 mL). The Pyrex tube was degassed by three freeze–pump–thaw cycles, sealed under vacuum, and heated at 180 °C for 3 d. After opening the tube, the solid was collected by filtration and washed with tetrahydrofuran and acetone. Dried in a vacuum oven at 80 °C for 24 h to obtain a fluffy red solid powder (20.6 mg, yield 92%).

**S1.15 Synthesis of PTTN-AO COF**

Ethanol (20 mL), NH_2_OH∙HCl (516.0 mg), COF PTTN-CN (85 mg) and triethylamine (120 mg) were mixed in a flask. Then, the above mixture was stirred at 82 °C for 24 h. Finally, the product COF-PDAN-AO was isolated by filtration, washed several times with Milli-Q water and ethanol, and dried under vacuum at 60 °C

**Section S2. Characterization Figures**


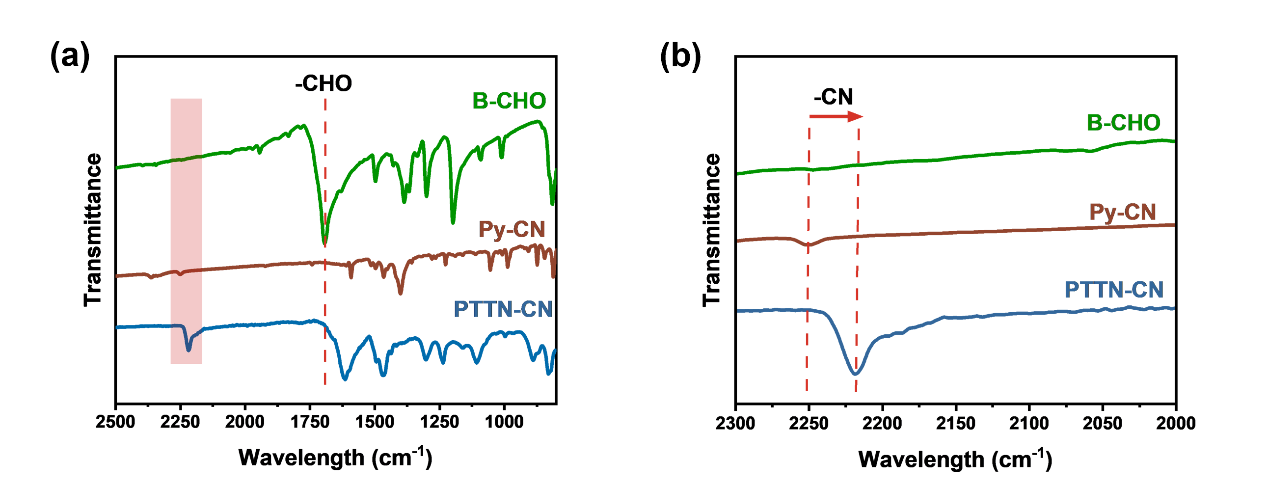


**Figure S2.** (a) FT-IR spectra of PTTN-CN and its precursors; (b) Range between the red region in (a).


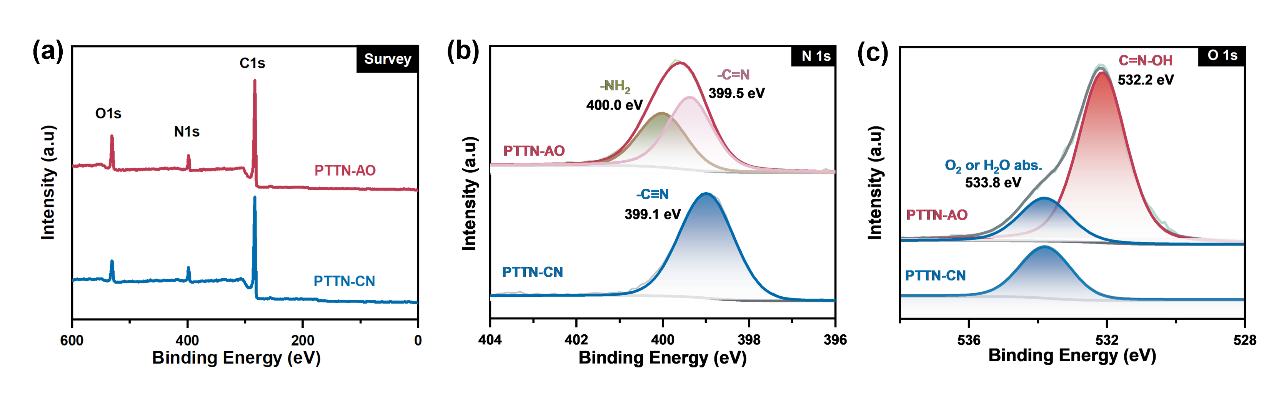


**Figure S3.** (a) XPS survey spectra for PTTN-CN and PTTN-AO; (b) N1s XPS spectra for PTTN-CN and PTTN-AO; (c) O 1s XPS spectra for PTTN-CN and PTTN-AO.


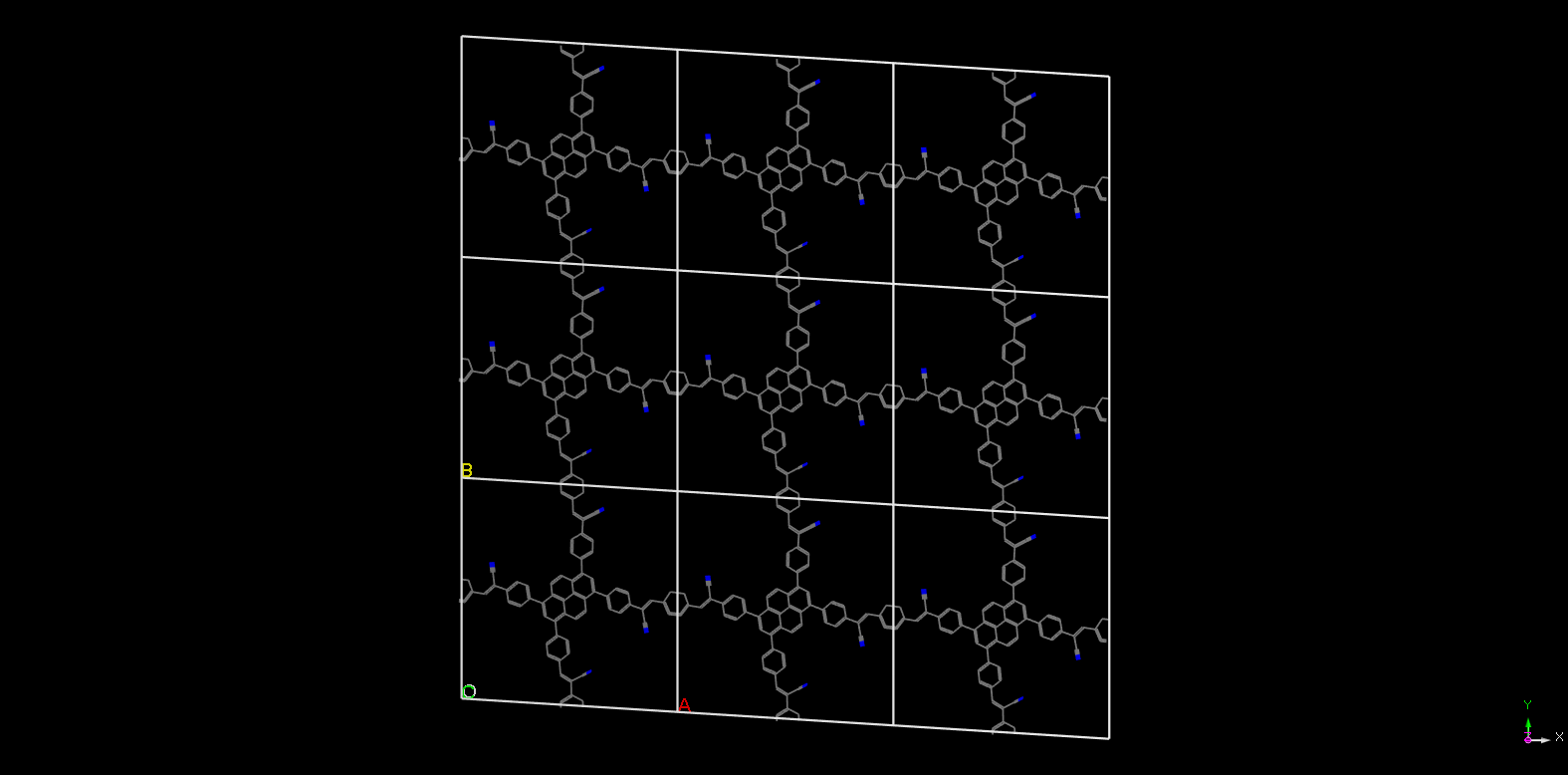


**Figure S4.** The optimized structure of PTTN-CN COF.


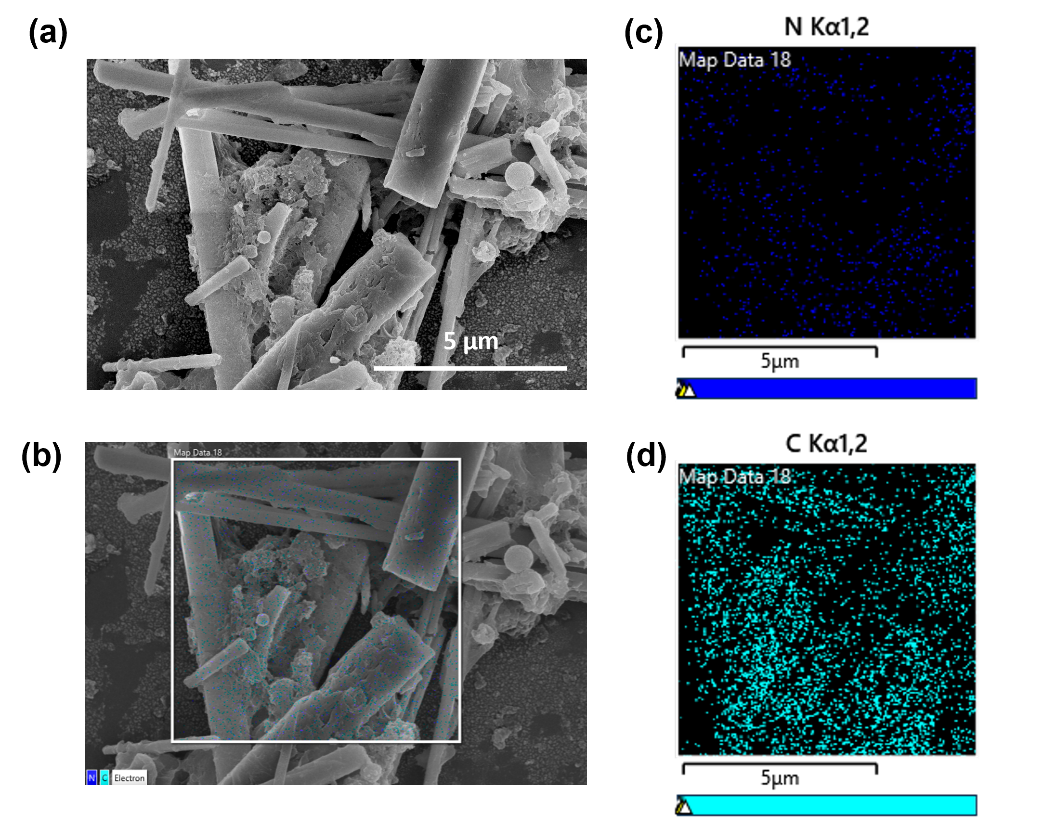


**Figure S5.** SEM images and EDS of PTTN-CN COF.


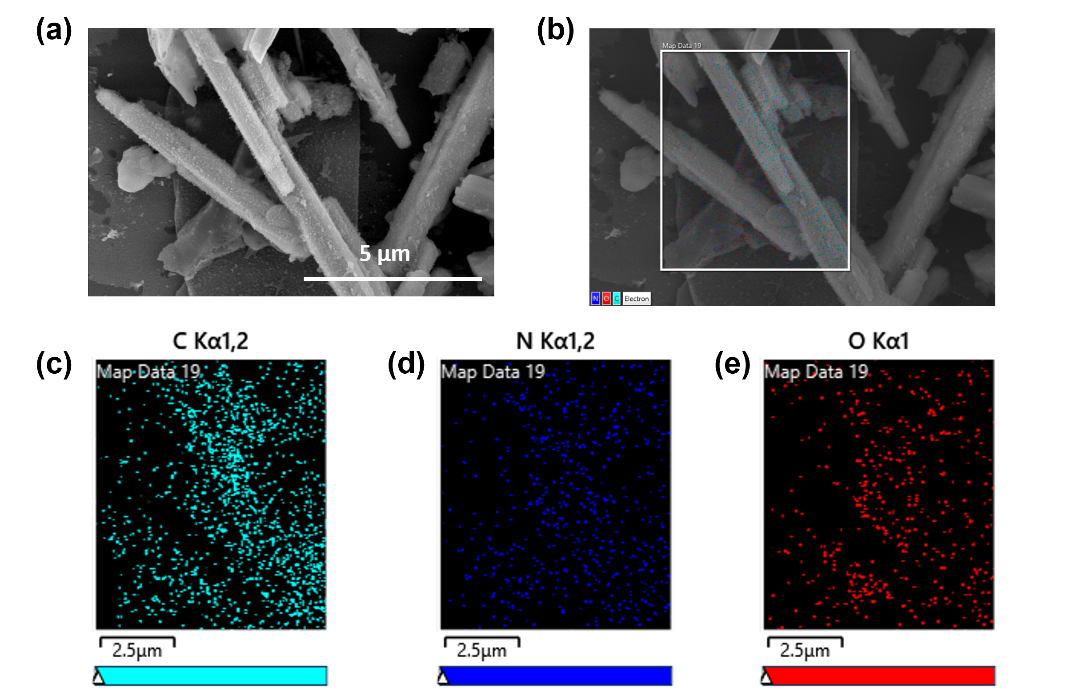


**Figure S6.** SEM images and EDS of PTTN-AO COF.


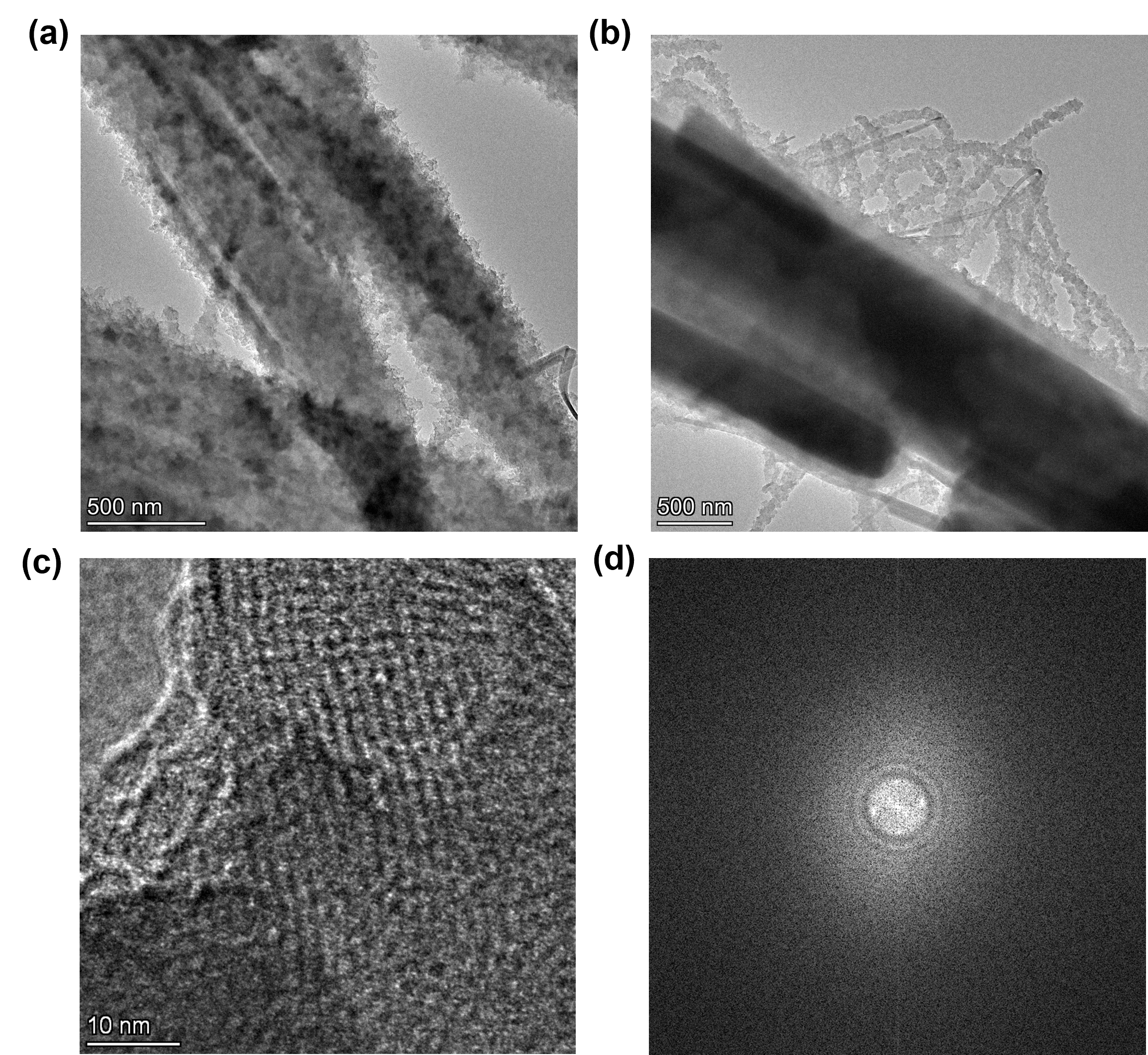


**Figure S7.** HR-TEM images and corresponding lattice fringes of (a) PTT-CN; (b, c, d) PTT-AO.


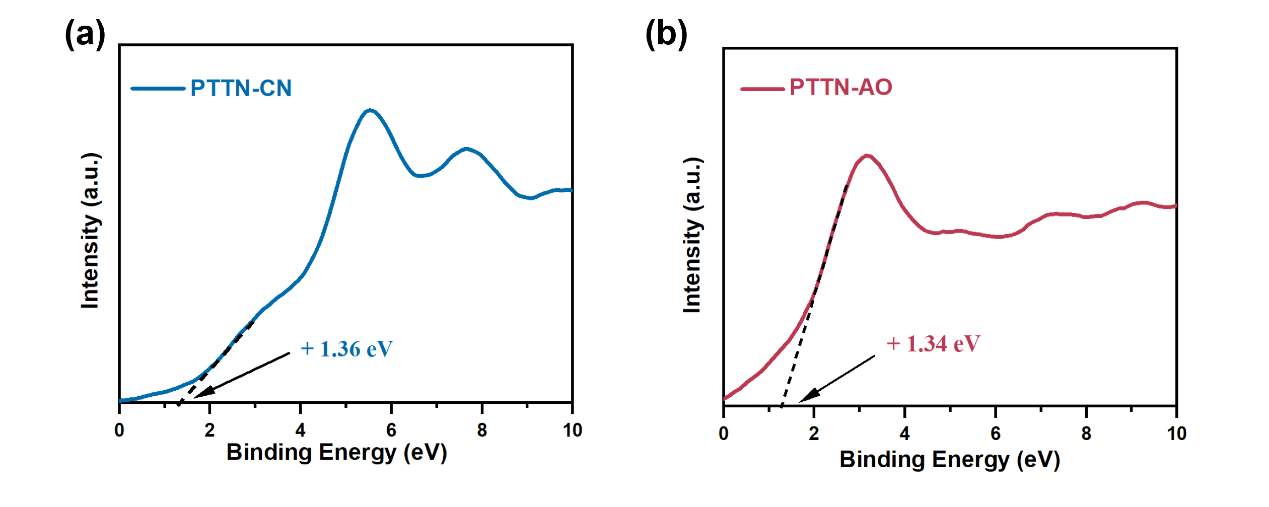


**Figure S8.** XPS-VB spectra of (a) PTTN-CN; and (b) PTTN-AO.

**
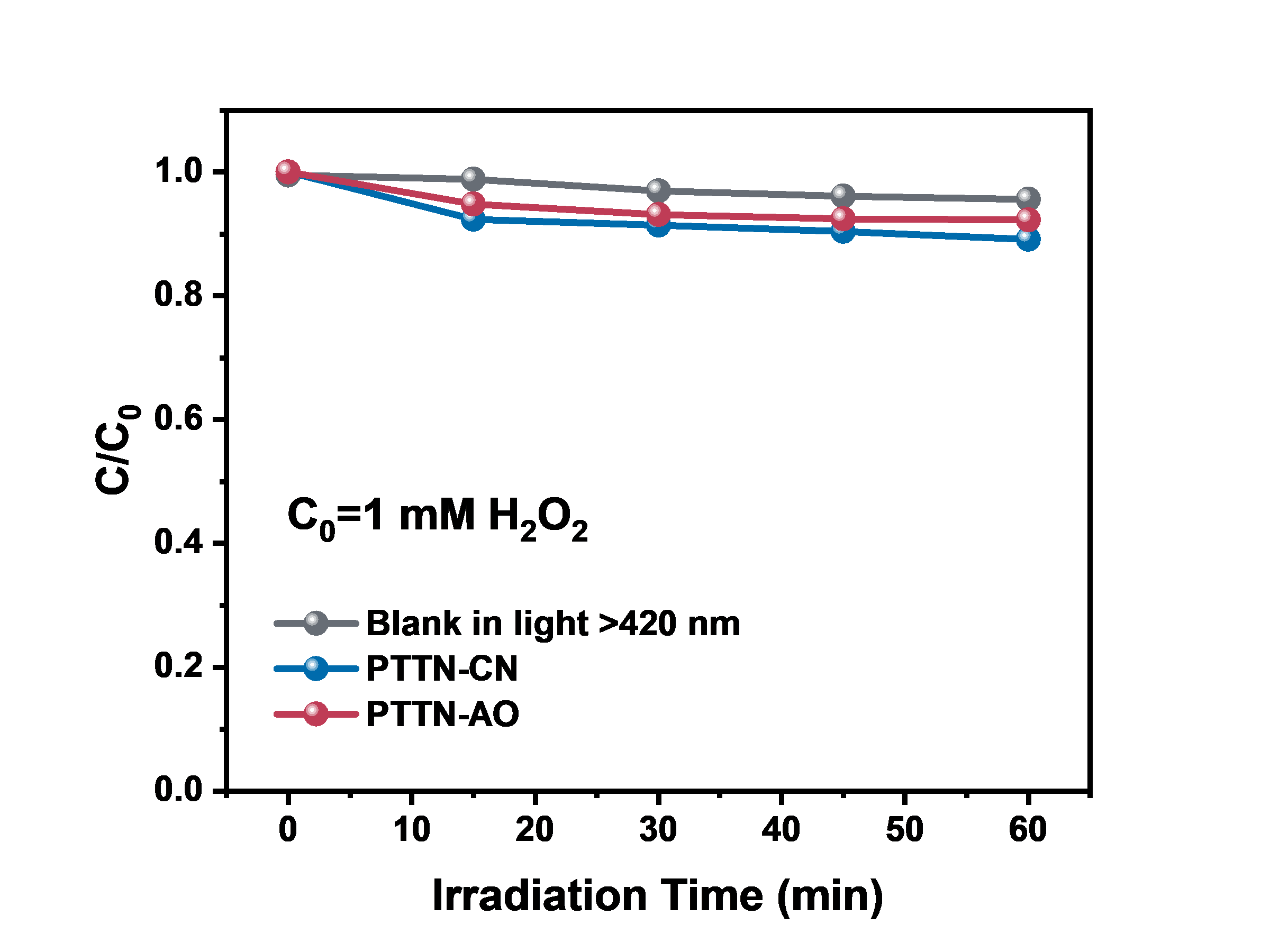
**

**Figure S9.** Decomposition of H_2_O_2_ over PTTN-CN and PTTN-AO in N_2_ atmosphere (λ > 420nm, 300 W Xenon lamp; 5 mg catalyst in 50 mL 1 mM H_2_O_2_ solution; 15 °C).

**
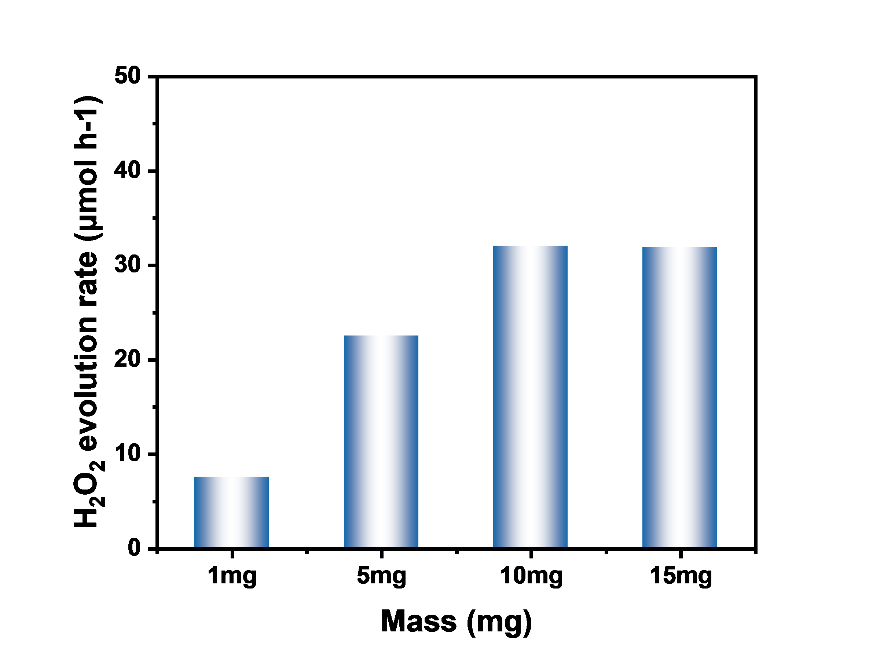
**

**Figure S10.** The photocatalytic H_2_O_2_ evolution rates as a function of the mass of PTTN-AO.

**
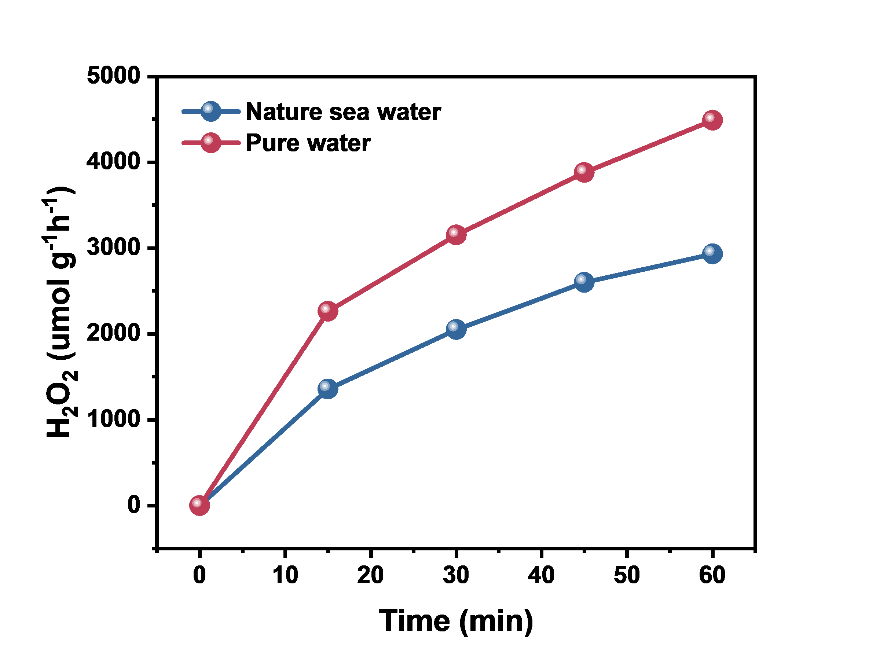
**

**Figure S11.** Photocatalytic H_2_O_2_ evolution by PTTN-AO under nature sea water.


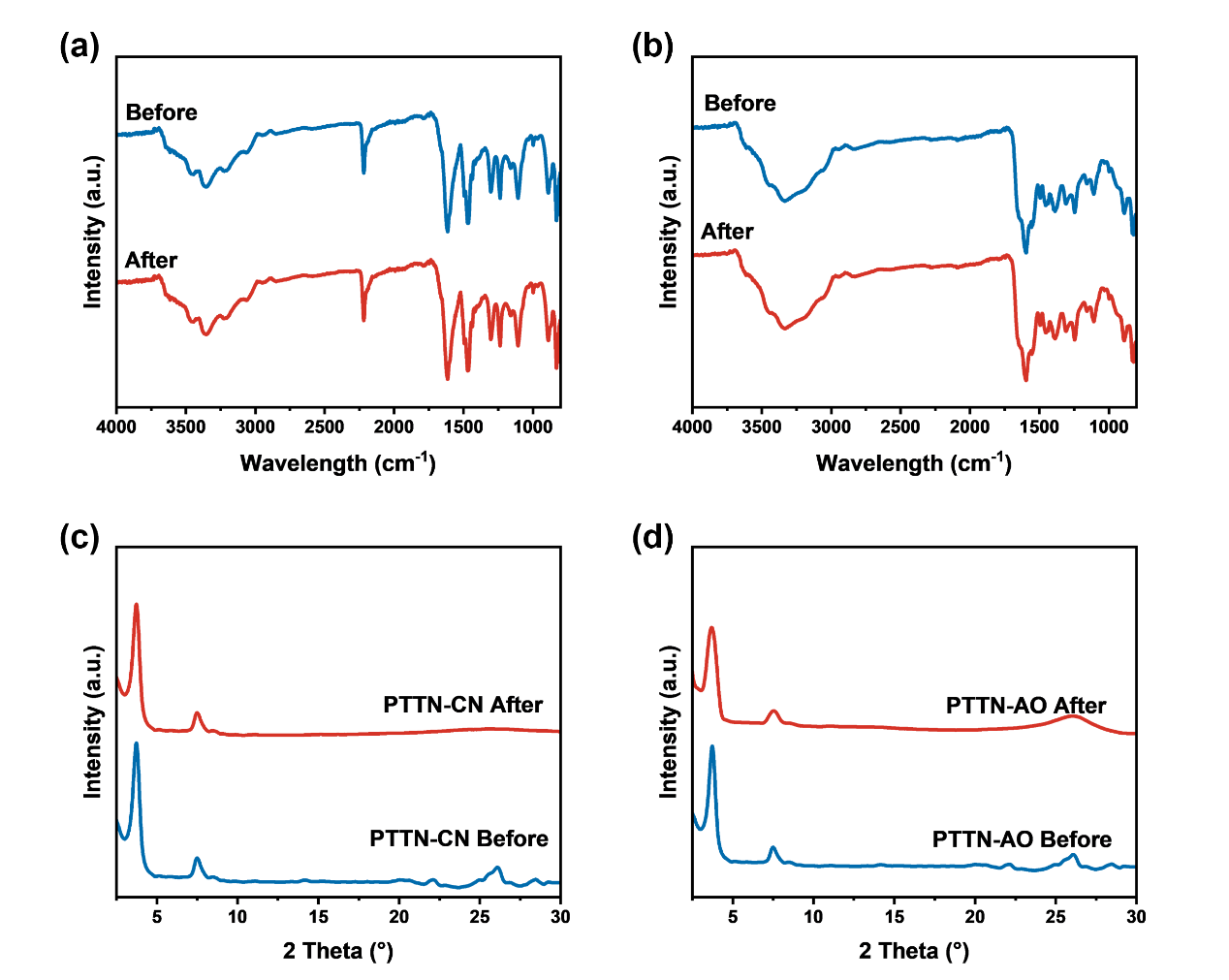


**Figure S12.** FT-IR spectra and PXRD pattern of the as-prepared and recycled PTTN-CN and PTTN-AO after the long-term continuous H_2_O_2_ photosynthesis experiment.

**
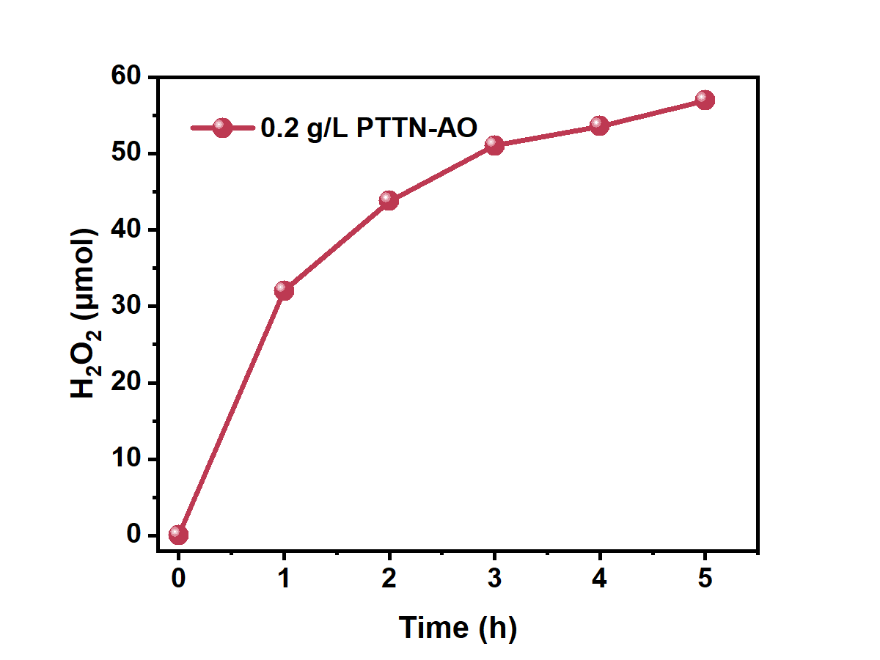
**

**Figure S13.** Long-term continuous H_2_O_2_ photosynthesis experiment of PTTN-AO.


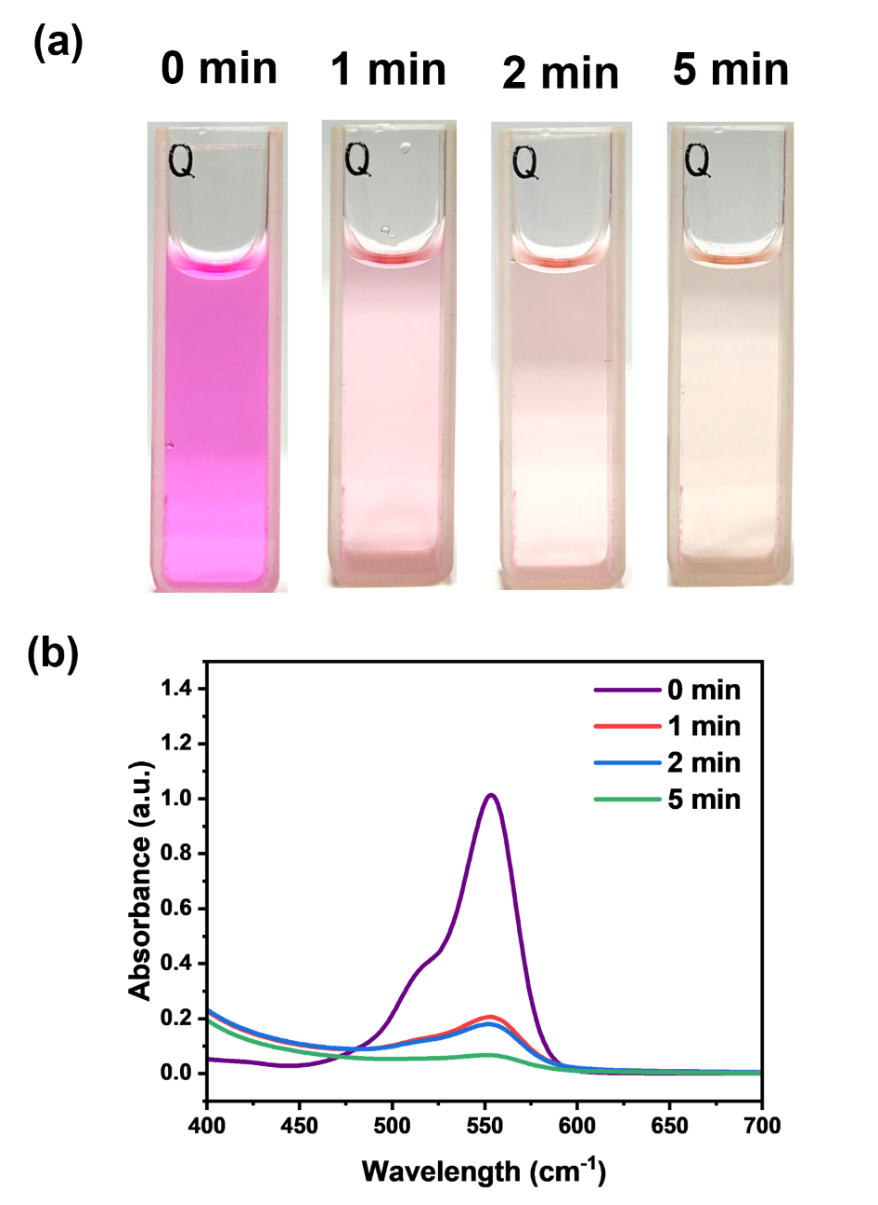


**Figure S14.** The RhB (10 mg L^−1^) decomposition using produced H_2_O_2_ solution *via* a Fenton reaction: (a) Color change with time; and (b) its corresponding absorbance.


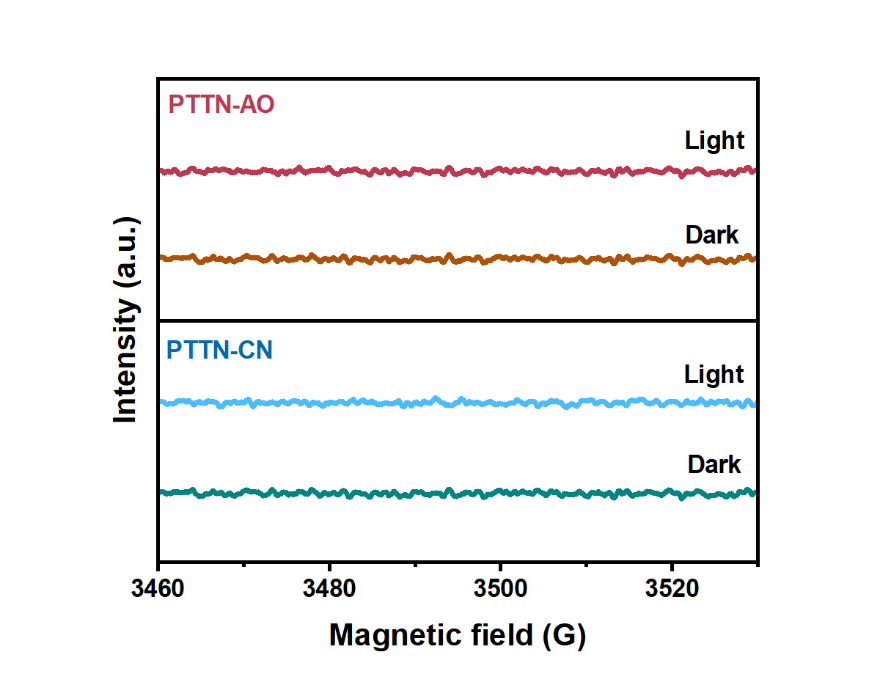


**Figure S15.** (a) DMPO-•OH EPR spectra of PTTN-CN and PTTN-AO under dark and light irradiation in 5,5-Dimethyl-1-pyrroline N-oxide (DMPO).


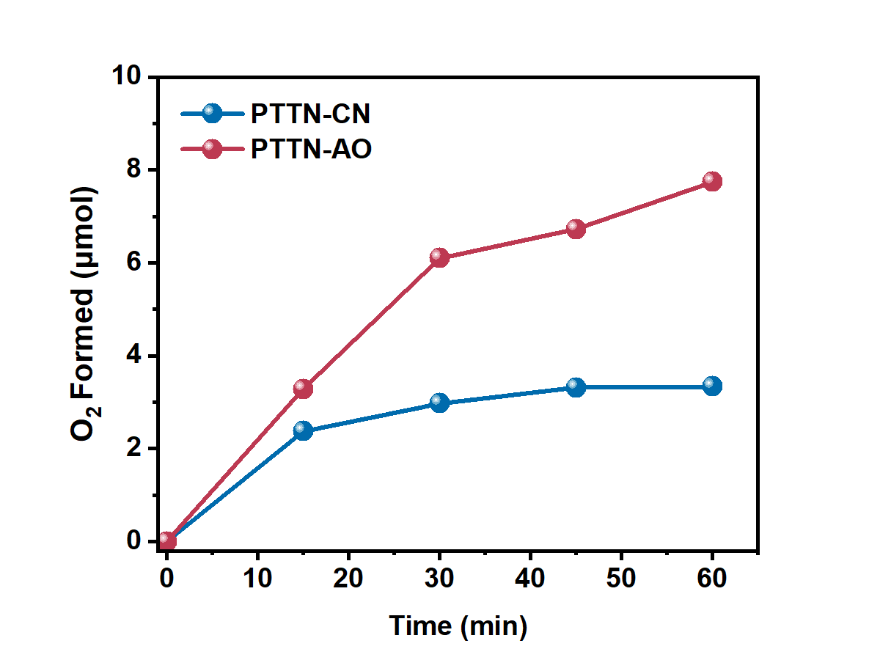


**Figure S16.** Time-dependent formation of O_2_ in AgNO_3_ aqueous solutions.


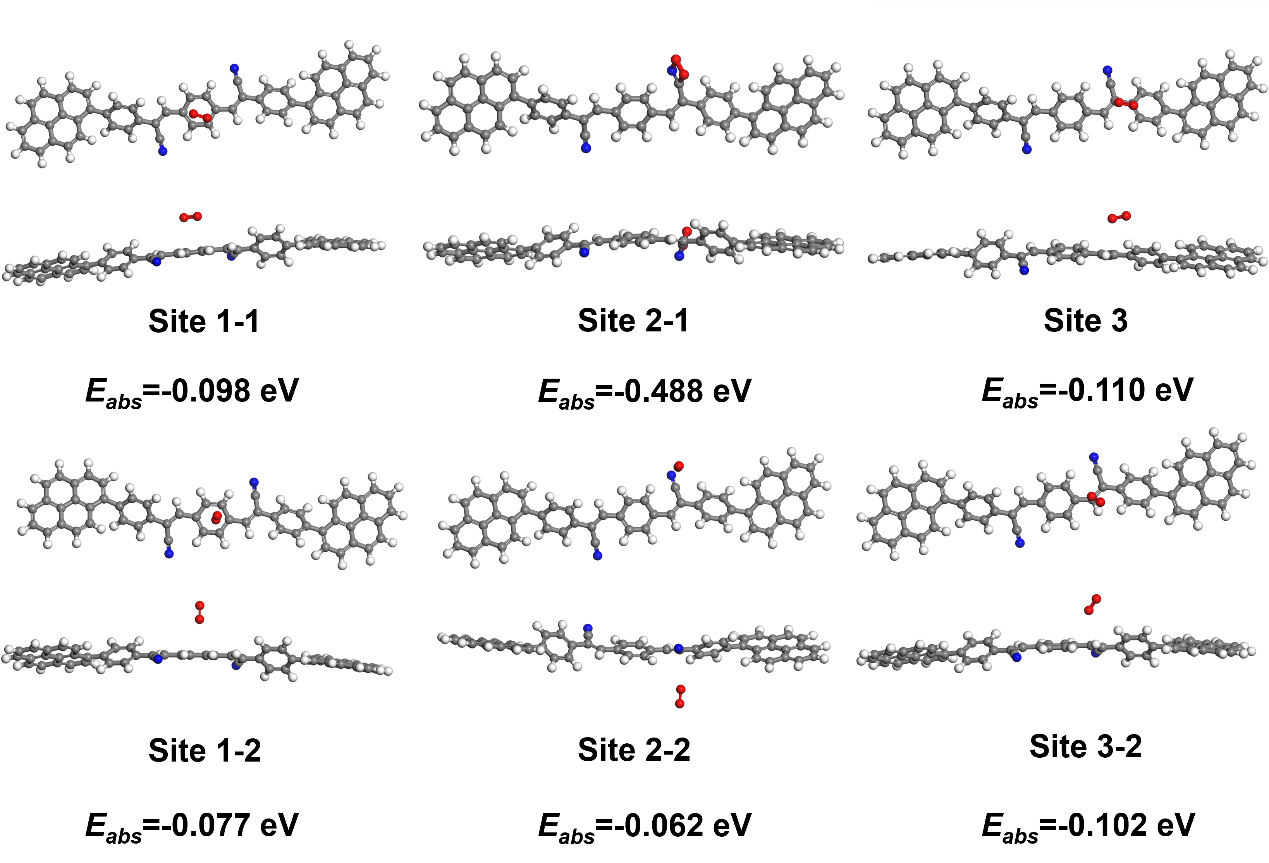


**Figure S17.** Different O_2_ adsorption configurations and adsorption energy for PTTN-CN.


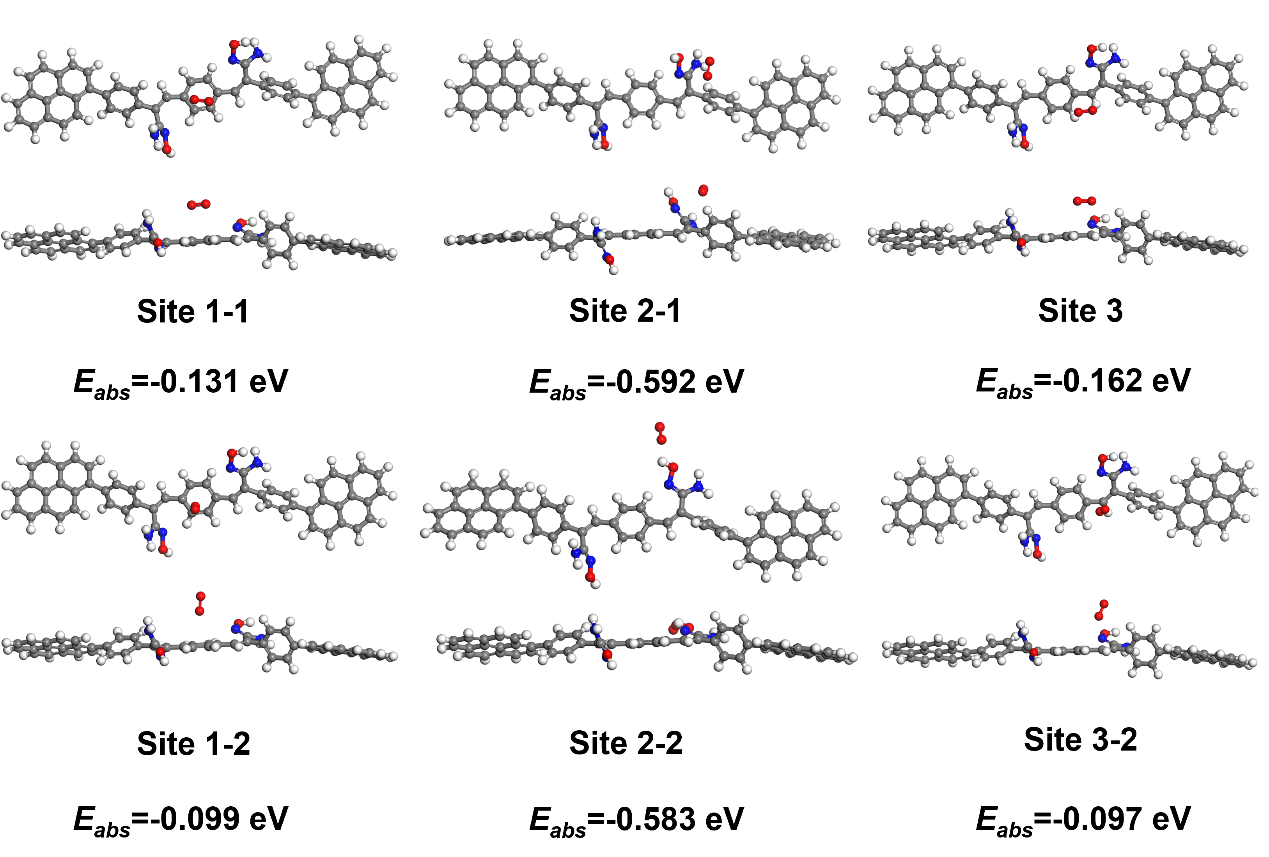


**Figure S18.** Different O_2_ adsorption configurations and adsorption energy for PTTN-AO.


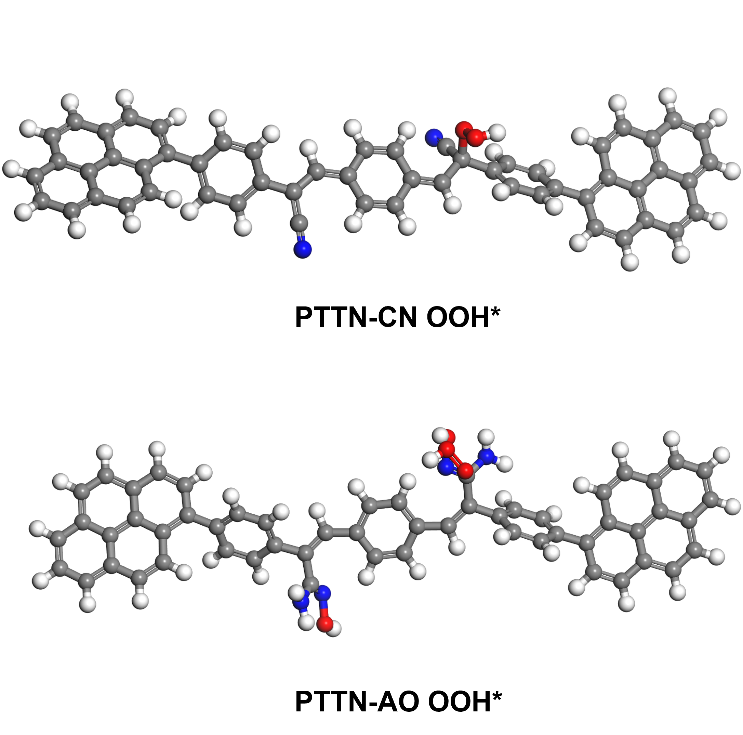


**Figure S19.** *OOH adsorption configurations for PTTN-CN COF and PTTN-AO COF.


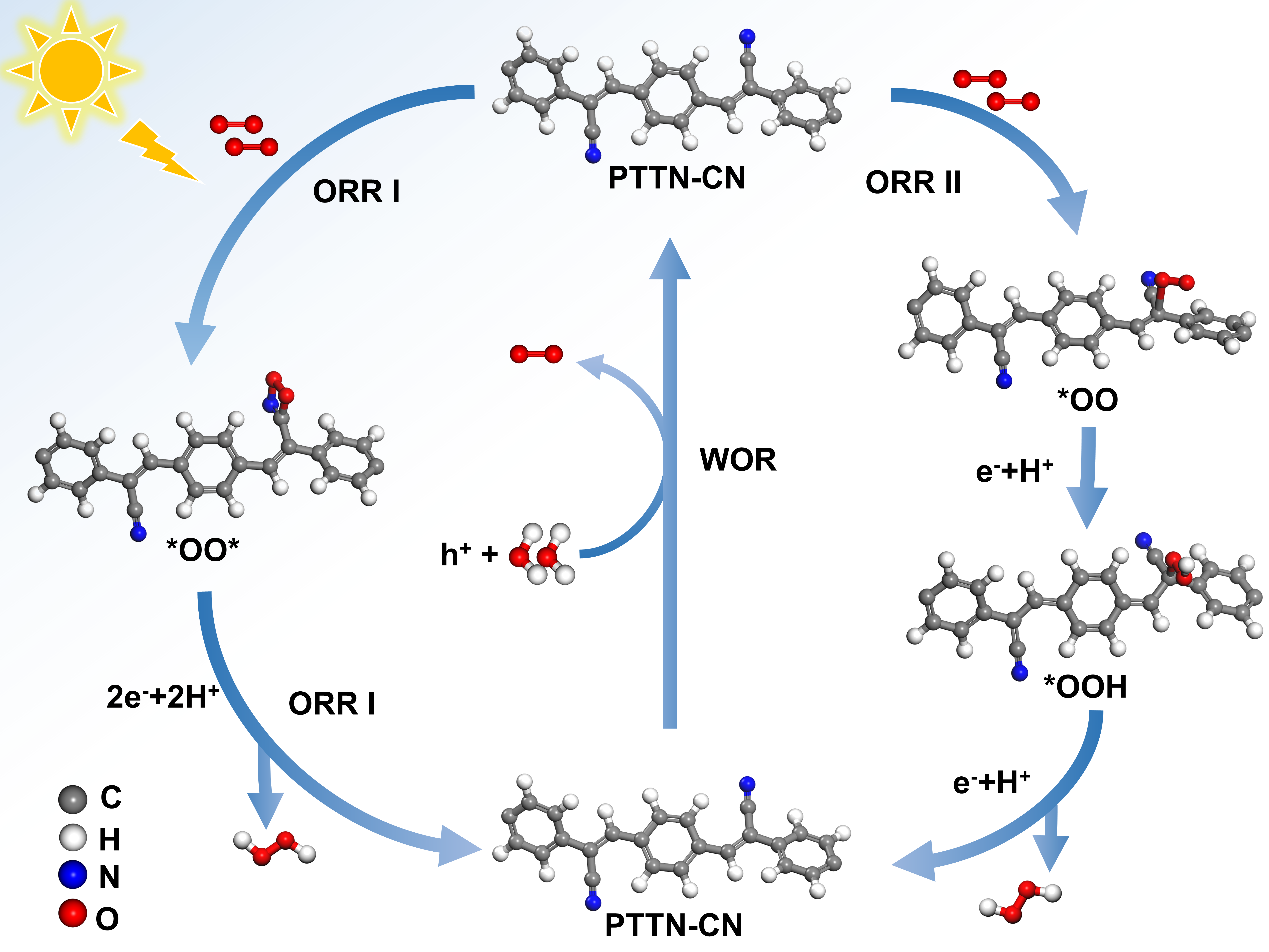


**Figure S20.** Proposed reaction mechanism toward H_2_O_2_ production on the PTTN-CN COF.

**Section S3. Supplementary Tables**

**Table S1**. Comparison of photocatalytic performances among recently reported COFs

| **Photocatalyst** | **H_2_O_2_ yield rate**  **μmol h^-1^ g^-1^** | **Irradiated**  **conditions** | | **Solvent** | ***SCC (%)*** | **Ref** |
| --- | --- | --- | --- | --- | --- | --- |
| PTTN-AO | 6024 | λ > 420 nm | H_2_O, O_2_ | | 0.61 | **This work** |
|  | 12900 | λ > 420 nm | Water: BA=9:1 | | - |  |
| CTF-BDDBN | 97.2 | λ > 420 nm | H_2_O | | 0.14 | *Adv. Mater.* **2020**, *32*, 1904433. |
| sonoCOF-F2 | 164 | λ >420 nm | H_2_O | | - | *J. Am. Chem. Soc.* 2022, 144, 9902 |
|  | 415 | λ >420 nm | Water: BA=9:1 | | - |  |
| **TF_50_-COF** | 1739 | λ > 420 nm | H_2_O: EtOH=9:1 | | 0.17 | *Angew. Chem. Int. Ed.* 2022, 61, e202202328 |
| **SO_3_H-COF** | 3015 | λ >420 nm | H_2_O, O_2_ | | 0.4 | *Angew. Chem. Int. Ed.* 2024, e202404077 |
| **Bpy-TAPT** | 4038 | λ > 420 nm | H_2_O, O_2_ | | 0.65 | *Appl. Catal. B: Environ.* **2023**, *331*, 122691. |
| **HEP-TAPT**-**COF** | 1705 | λ > 420 nm | H_2_O, O_2_ | | 0.65 | *Angew. Chem. Int. Ed.* 2023, 62, e202217479 |
| **TD-COF** | 3364 | λ > 420 nm | H_2_O, natural seawater | | 0.15 | *Angew. Chem. Int. Ed.* 2023, 62, e202309624 |
| **Py-Da-COF** | 1242 | λ > 420 nm | H_2_O, O_2_ | | 0.09 | *Angew. Chem. Int. Ed.* 2023, 62, e202216719 |
| **TDB-COF** | 723.5 | λ > 420 nm | H_2_O, air | | 0.39 | *Appl. Catal. B: Environ.* **2023**, *334*, 122862. |
| **COF-2CN** | 4858 | λ > 420 nm | H_2_O, air | | 0.6 | *Angew. Chem. Int. Ed. 2024, 63, e202318562* |
| **o-COF-TpPzda** | 4396 | λ > 420 nm | H_2_O, O_2_ | | 0.46 | *Angew. Chem. Int. Ed. 2024, 63, e202404077* |
| **TaptBtt** | 1407 | λ > 420 nm | H_2_O, O_2_ | | 0.296 | *Nat. Commun.* **2023**, *14*, 5238. |
| **DE7-M** | 2216 | λ > 420 nm | H_2_O | | 0.28 | *J. Am. Chem. Soc.* **2021**, *143*, 19287-19293. |
| **TTF-BT-COF** | 2760 | λ > 420 nm | H_2_O, O_2_ | | 0.49 | *Angew. Chem. Int. Ed.* **2023**, *62*, e202218868. |
| **FS-COFs** | 1506.1 | λ > 420 nm | H_2_O, O_2_ | | - | *Angew. Chem. Int. Ed.* 2023, 62, e202305355 |
|  | 3904 | λ > 420 nm |  |  | - |  |
| COF-TfpBpy | 695 | λ > 420 nm | H_2_O, air | | 0.57 | *Angew. Chem. Int. Ed.* **2022**, *61*, e202200413. |
|  | 1313 | λ>300 | H_2_O, air | | 1.08 |  |
| **FS-OHOMe-COF** | 2200 | λ > 420 nm | H_2_O, air | | 0.58 | *Angew. Chem. Int. Ed.* 2024, 63, e202403926 |
| **DMCR-1NH** | 2588 | 420-700 nm | Water:BA=9:1 | | - | *J. Am. Chem. Soc.* 2023, 145, 2975 |
| **CoPc-BTM-COF** | 2096 | λ > 400 nm | H_2_O: EtOH=9:1 | | - | *J. Am. Chem. Soc.* 2022, 144, 21328 |

**Table S2. Fractional atomic coordinates and the unit cell of PTTN-CN**

| PTTN-CN | | Space group *= P1*  a = 3.66 Å, b = 23.37 Å, c = 23.83 Å,  𝛼 = 86.47°, 𝛽 = 89.00°, 𝛾 = 91.91° | |
| --- | --- | --- | --- |
| C1 | 0.55729 | -0.39765 | -0.03426 |
| C2 | 0.51123 | -0.43438 | -0.01012 |
| C3 | 0.51809 | -0.49298 | 0.02179 |
| C4 | 0.57062 | -0.51480 | 0.04003 |
| C5 | 0.61635 | -0.47805 | 0.02632 |
| C6 | 0.60831 | -0.42046 | -0.01501 |
| C7 | 0.45909 | -0.41399 | -0.02641 |
| C8 | 0.41430 | -0.45026 | -0.01033 |
| C9 | 0.41984 | -0.50830 | 0.01647 |
| C10 | 0.47247 | -0.52988 | 0.03162 |
| C11 | 0.47986 | -0.58824 | 0.05472 |
| C12 | 0.53190 | -0.60851 | 0.07426 |
| C13 | 0.57622 | -0.57263 | 0.06592 |
| C14 | 0.37418 | -0.54548 | 0.03143 |
| C15 | 0.38309 | -0.60305 | 0.05603 |
| C16 | 0.43487 | -0.62522 | 0.06326 |
| C17 | 0.55592 | -0.33537 | -0.08917 |
| C18 | 0.67322 | -0.49630 | 0.05041 |
| C19 | 0.44065 | -0.68665 | 0.06809 |
| C20 | 0.31704 | -0.52729 | 0.01502 |
| C21 | 0.30806 | -0.47112 | -0.11345 |
| C22 | 0.25478 | -0.45537 | -0.13467 |
| C23 | 0.20880 | -0.49553 | -0.02979 |
| C24 | 0.21748 | -0.55182 | 0.09756 |
| C25 | 0.27084 | -0.56742 | 0.11880 |
| C26 | 0.39339 | -0.72543 | 0.26252 |
| C27 | 0.39951 | -0.78323 | 0.26990 |
| C28 | 0.45295 | -0.80302 | 0.08458 |
| C29 | 0.49933 | -0.76413 | -0.12272 |
| C30 | 0.49324 | -0.70685 | -0.13040 |
| C31 | 0.68360 | -0.55301 | 0.14860 |
| C32 | 0.73679 | -0.56875 | 0.17197 |
| C33 | 0.78127 | -0.52803 | 0.10017 |
| C34 | 0.77118 | -0.47124 | 0.00432 |
| C35 | 0.71796 | -0.45569 | -0.01968 |
| C36 | 0.60669 | -0.30256 | -0.30498 |
| C37 | 0.60814 | -0.24426 | -0.34661 |
| C38 | 0.55891 | -0.21686 | -0.17263 |
| C39 | 0.50777 | -0.24953 | 0.04321 |
| C40 | 0.50633 | -0.30826 | 0.08479 |
| C41 | 0.56261 | -0.15476 | -0.20855 |
| C42 | 0.15237 | -0.47878 | -0.05687 |
| C43 | 0.45888 | -0.86416 | 0.09729 |
| C44 | 0.50880 | -0.95193 | 0.04746 |
| C45 | 0.56151 | -0.97698 | -0.04323 |
| C46 | 0.50745 | -0.89104 | 0.06463 |
| C47 | 0.83783 | -0.54473 | 0.12461 |
| C48 | 0.95843 | -0.56024 | 0.16324 |
| C49 | 0.93496 | -0.50823 | 0.10465 |
| C50 | 0.96730 | -0.45815 | 0.08209 |
| C51 | 0.87924 | -0.50275 | 0.09386 |
| C52 | 0.45873 | -0.98540 | 0.11378 |
| C53 | 0.51667 | -0.12777 | -0.02628 |
| C54 | 0.10747 | -0.52017 | 0.04423 |
| C55 | 0.05167 | -0.51444 | 0.03562 |
| C56 | 0.51476 | -0.06698 | -0.00905 |
| C57 | 0.46176 | -0.04260 | 0.08810 |
| C58 | 0.56439 | -0.03367 | -0.06975 |
| C59 | 0.03211 | -0.46251 | -0.11126 |
| C60 | 0.01539 | -0.56307 | 0.14721 |
| C61 | 0.84873 | 0.39621 | 0.17930 |
| N62 | 0.85735 | 0.34895 | 0.22281 |
| C63 | 0.61657 | 0.87570 | -0.44708 |
| N64 | 0.65969 | 0.89984 | -0.63896 |
| C65 | 0.14527 | 0.57980 | -0.18728 |
| N66 | 0.13979 | 0.62667 | -0.29242 |
| C67 | 0.55935 | 0.13835 | 0.05253 |
| N68 | 0.60073 | 0.16170 | 0.04464 |

**Section S4. Supporting References**

[1] a) K. J. G, Furthmüller., *Computational Materials Science* **1996**, *6*, 15-50; b) K. J. G, Hafner., *Phys. Rev. B* **1993**, *48*, 13115.

[2] C. Rostgaard, *Physics* **2009**, *62*, 11556-11570.

[3] P. K. John P, Burke; Matthias ,Ernzerhof, *Physical Review Letters* **1998**, *77*, 3865-3868.

[4] a) V. X. Wang, N.; Liu, J.-C.; Tang, G.; Geng, W. T., *arXiv* **1908**, *6*, 08269; b) W. Yi, G. Tang, X. Chen, B. Yang, X. Liu, *Computer Physics Communications* **2020**, *257*.

[5] Y. Wang, Y. Z. Cheng, K. M. Wu, D. H. Yang, X. F. Liu, X. Ding, B. H. Han, *Angew. Chem. Int. Ed.* 2023, 62, e202310794.
